# Neuroimaging techniques, gene therapy, and gut microbiota: frontier advances and integrated applications in Alzheimer’s Disease research

**DOI:** 10.3389/fnagi.2024.1485657

**Published:** 2024-12-03

**Authors:** Haitao Wang, Chen Shi, Ling Jiang, Xiaozhu Liu, Rui Tang, Mingxi Tang

**Affiliations:** ^1^School of Basic Medicine, Southwest Medical University, Luzhou, Sichuan, China; ^2^The School of Clinical Medical Sciences, Southwest Medical University, Luzhou, Sichuan, China; ^3^Department of Gynaecology, Shengjing Hospital of China Medical University, Shenyang, Liaoning, China; ^4^Department of Anorectal, The Affiliated Traditional Chinese Medicine Hospital, Southwest Medical University, Luzhou, China; ^5^Emergency and Critical Care Medical Center, Beijing Shijitan Hospital, Capital Medical University, Beijing, China; ^6^Department of Pathology, Yaan People’s Hospital (Yaan Hospital of West China Hospital of Sichuan University), Yaan, Sichuan, China

**Keywords:** Alzheimer’s Disease, neuroimaging techniques, gene therapy, gut microbiota, frontier advances, interdisciplinary research

## Abstract

Alzheimer’s Disease (AD) is a neurodegenerative disorder marked by cognitive decline, for which effective treatments remain elusive due to complex pathogenesis. Recent advances in neuroimaging, gene therapy, and gut microbiota research offer new insights and potential intervention strategies. Neuroimaging enables early detection and staging of AD through visualization of biomarkers, aiding diagnosis and tracking of disease progression. Gene therapy presents a promising approach for modifying AD-related genetic expressions, targeting amyloid and tau pathology, and potentially repairing neuronal damage. Furthermore, emerging evidence suggests that the gut microbiota influences AD pathology through the gut-brain axis, impacting inflammation, immune response, and amyloid metabolism. However, each of these technologies faces significant challenges, including concerns about safety, efficacy, and ethical considerations. This article reviews the applications, advantages, and limitations of neuroimaging, gene therapy, and gut microbiota research in AD, with a particular focus on their combined potential for early diagnosis, mechanistic insights, and therapeutic interventions. We propose an integrated approach that leverages these tools to provide a multi-dimensional framework for advancing AD diagnosis, treatment, and prevention.

## 1 Introduction

Alzheimer’s Disease (AD) represents a complex neurodegenerative disorder characterized by progressive cognitive decline and neurodegeneration, constituting the predominant form of dementia in aging populations (Scheltens et al., 2021). Marked by cognitive decline and neuronal damage, the disease is primarily linked to the abnormal accumulation of amyloid-beta (Aβ) and the formation of neurofibrillary tangles (NFTs) (Yang et al., 2022; Taylor et al., 2023; Ashrafian et al., 2021; Otero-Garcia et al., 2022). However, despite significant advances in neuroscience, the pathogenesis of AD remains incompletely understood.

Currently, the diagnostic landscape for AD remains critically challenging. Current diagnostic practices primarily depend on clinical evaluations and neuropsychological assessments, which often fail to capture the subtle, early pathological changes associated with the disease (Atri, 2019; El Haj et al., 2023). This limitation highlights the urgent need for more effective diagnostic tools. Although symptomatic treatments, including cholinesterase inhibitors and *N*-methyl-d-aspartate receptor antagonists, can provide temporary cognitive benefits, they do not halt or reverse the disease’s progression (Lista et al., 2023; Companys-Alemany et al., 2022). Thus, the exploration of novel approaches is critical.

In recent years, neuroimaging techniques, gene therapy, and gut microbiota have emerged as three hotspots in research, showing significant promise in the treatment and prevention of AD, thus attracting widespread attention and exploration. Neuroimaging techniques can visualize early biomarkers and specific imaging changes associated with AD, thereby enhancing diagnostic accuracy and sensitivity. For example, imaging modalities can detect Aβ deposition and NFTs distribution, allowing for the localization of affected brain regions and monitoring of neuronal activity and metabolic changes. Gene therapy could intervene in the pathogenesis of AD by altering the expression or function of AD-related genes. Strategies may include reducing Aβ production, enhancing its clearance, inhibiting the formation of NFTs, promoting their degradation, and repairing damaged neurons, as well as modulating immune and inflammatory responses (Bhardwaj et al., 2022; Griciuc et al., 2020). Additionally, the gut microbiota may influence the pathogenesis of AD through the gut-brain axis, affecting Aβ metabolism, modulating immune responses, and influencing neurotransmitter and neurotrophic factor levels, which could contribute to the prevention of AD development (Kesika et al., 2021; Das and Ganesh, 2023).

This article aims to review the comprehensive applications and progress of neuroimaging techniques, gene therapy, and gut microbiota in AD research. We will analyze their respective advantages and limitations, exploring the potential for synergistic effects among these approaches. Such integration could open new pathways for AD research and treatment, paving the way for individualized and comprehensive therapeutic strategies.

## 2 Applications of neuroimaging techniques in AD research

### 2.1 Overview of neuroimaging techniques

Neuroimaging techniques refer to the use of various imaging methods, such as Magnetic Resonance Imaging (MRI), Positron Emission Tomography (PET), and Single Photon Emission Computed Tomography (SPECT), to observe and analyze structural and functional changes in the nervous system (Risacher and Saykin, 2021).

Among these, MRI has emerged as one of the most commonly used and versatile methods in neuroimaging. Specifically, there are several specialized MRI techniques that have revolutionized AD research: It leverages magnetic fields and radiofrequency pulses to produce high-resolution anatomical images (Chen and Steckner, 2017). Recent advancements in MRI enable it to reveal detailed microstructural changes, particularly relevant to AD, through various specialized techniques and sequences. For example, Diffusion Tensor Imaging (DTI) and Diffusion Weighted Imaging (DWI) provide insights into white matter integrity and connectivity by measuring water molecule diffusion along neural pathways, helping to detect axonal and myelin damage associated with AD (Leandrou et al., 2018). Susceptibility-Weighted Imaging (SWI) enhances visualization of iron deposits in brain regions affected by AD (Rashid et al., 2021), while Arterial Spin Labeling (ASL) non-invasively measures cerebral blood flow (Zhang et al., 2021). Furthermore, functional MRI (fMRI) monitors changes in brain oxygen levels to study brain function (Sheline and Raichle, 2013). Through Blood Oxygen Level Dependent (BOLD) imaging, fMRI evaluates functional connectivity within brain networks, shedding light on alterations in AD that affect cognitive processes and network synchrony (Arbabyazd et al., 2023).

Beyond MRI, PET, and SPECT are also widely used in AD research, providing quantitative information about neuronal function and metabolism (Xiang et al., 2021; Colloby et al., 2016). PET uses radiolabeled tracers such as fluorodeoxyglucose (FDG) to assess glucose metabolism (Park et al., 2023), Aβ tracers to measure amyloid deposits (Chouliaras et al., 2022), and tau-specific tracers to reveal NFTs (Wagatsuma et al., 2023), thus enabling direct evaluation of AD’s hallmark pathology. Similarly, SPECT offers valuable insights into regional cerebral blood flow and dopamine receptor activity, which are disrupted in AD. MRS enables the measurement of metabolic compounds such as *N*-acetylaspartate, glutamate, and myo-inositol, which serve as indicators of neuronal health, neuroinflammation, and glial activity, providing insights into AD-related metabolic disturbances (Chaney et al., 2021). EEG, a cost-effective technique, records electrical activity, reflecting synaptic function and neural oscillations that correlate with cognitive impairment in AD (Gaubert et al., 2019). Near-Infrared Spectroscopy (NIRS), although less frequently used in AD, enables non-invasive monitoring of cortical oxygenation changes in real time, with applications in functional connectivity studies (Canova et al., 2012).

### 2.2 The application of neuroimaging techniques in AD research and clinical practice

#### 2.2.1 Early diagnosis and staging of AD

Structural neuroimaging techniques, including CT and MRI, reveal morphological changes in the brains of AD patients, such as widened cerebral sulci, enlarged ventricles, and atrophy of the hippocampus and entorhinal cortex (Jang et al., 2022; Yang et al., 2021). MRI, particularly with high-resolution T1-weighted imaging (Frisoni, 2001), can visualize subtle changes in the hippocampus and temporal lobe regions associated with early AD (Hampel et al., 2002; De Santi et al., 2001; Josephs et al., 2008). DTI and DWI assess white matter integrity and microstructural changes (Jin et al., 2017; Dou et al., 2020), while MRI can quantify gray matter atrophy rates through volumetric analysis (Upadhyay et al., 2016; Rahman et al., 2020). Additionally, multi-parameter MRI enables simultaneous measurement of brain atrophy, white matter changes, and vascular integrity, enhancing diagnostic sensitivity for early AD (Zhang and Liu, 2018).

Integration and machine learning improve the accuracy and sensitivity of early diagnosis and staging (Chételat, 2018). Multimodal integration combines MRI, PET, and MRS to correlate structural and functional data, providing an enriched view of AD progression. For instance, Sheng et al. (2024) proposed a multimodal machine learning framework that integrates various neuroimaging techniques with biomarkers to utilize complementary multimodal data for enhancing AD diagnosis (Table 1).

**Table 1:** Overview of neuroimaging techniques in Alzheimer’s Disease research and clinical practice.

Neuroimaging technique	Clinical application	Advantages	Limitations	References
MRI	Structural imaging of brain atrophy in AD	High spatial resolution; non-invasive; widely available	Limited in detecting early changes; requires patient compliance	Risacher and Saykin (2021), Jang et al. (2022), Yang et al. (2021), Upadhyay et al. (2016), Rahman et al. (2020), and Zhang and Liu (2018)
fMRI	Functional connectivity studies in AD	Real-time monitoring of brain activity; assesses cognitive processes	Susceptible to motion artifacts; indirect measure of neuronal activity	Sheline and Raichle (2013) and Arbabyazd et al. (2023)
PET	Assessment of amyloid and tau pathology	Provides quantitative measures of specific biomarkers	High operational costs; radiation exposure; limited accessibility	Park et al. (2023), Chouliaras et al. (2022), and Wagatsuma et al. (2023)
SPECT	Evaluation of cerebral blood flow and neurotransmitter activity	Useful for assessing perfusion changes; relatively easy to perform	Lower resolution than PET; challenges in quantitative analysis	Herholz (2011), Sala et al. (2021), Marcolini et al. (2022), David et al. (2008), and Depboylu et al. (2013)
DTI and DWI	Assessment of white matter integrity	Detects microstructural changes; sensitive to axonal injury	Interpretation can be complex; influenced by other pathology	Leandrou et al. (2018), Jin et al. (2017), and Dou et al. (2020)
MRS	Measurement of metabolic compounds	Non-invasive; provides insight into neuronal health and metabolism	Limited spatial resolution; specific expertise required for analysis	Chaney et al. (2021) and Yeh et al. (2018)
EEG	Monitoring electrical activity associated with cognition	Cost-effective; excellent temporal resolution	Poor spatial resolution; difficult to localize activity	Gaubert et al. (2019)
NIRS	Non-invasive monitoring of cortical oxygenation changes	Real-time data acquisition; useful for functional connectivity studies	Less commonly used; limited depth of penetration	Canova et al. (2012)

#### 2.2.2 A biomarker development and validation

Neuroimaging techniques have revolutionized our ability to detect and monitor AD biomarkers *in vivo*, offering a comprehensive multi-modal approach to understanding AD pathology. These techniques can be broadly categorized into structural, functional, and molecular imaging modalities, each providing distinct yet complementary information about AD pathophysiology:

Positron Emission Tomography is a powerful technique, allowing for the visualization of AD-specific pathological processes (Wang et al., 2023). Most significantly, PET imaging with specific tracers, such as Aβ ligands and tau ligands, enables the direct and quantitative measurement of Aβ and tau tangles (Ruan and Sun, 2023; Chandra et al., 2019). Furthermore, fluorine-18 labeled FDG-PET is used to evaluate brain glucose metabolism, which serves as a proxy for neuronal activity, highlighting regions of hypometabolism that are frequently associated with AD (Albert et al., 2011). Levin et al. (2021) have used FDG-PET as a sensitive molecular imaging biomarker to explore data-driven subtypes of neurodegenerative changes in AD, identifying three main subtypes of metabolic decline. Another tracer, 18F-FEBMP, has been used by Ji et al. (2021) to assess neuroinflammation in AD, finding it to be an ideal PET ligand for detecting neuroinflammation associated with AD.

In terms of biomarker development, MRI can reveal the connectivity and integration of brain structures and functions and their relationships with cognitive reserve, cognitive training, sleep quality, etc. (Choe et al., 2019; Thams et al., 2020). Structural MRI (sMRI) using T1-weighted imaging enables precise measurements of brain atrophy patterns, with studies showing that medial temporal lobe atrophy can predict conversion from mild cognitive impairment to AD with 80–85% accuracy (Blamire, 2018). Specific sequences or techniques, such as DTI, fMRI, and MRS, are used to assess changes in neuronal connections, brain function, and metabolism in AD. A systematic review based on DTI showed that AD patients mainly exhibit extensive microstructural damage, structural discontinuities, and topological abnormalities in areas like the corpus callosum, cingulum, and medial temporal lobe, including the hippocampus and cingulate. Advanced diffusion imaging techniques, particularly neurite orientation dispersion and density imaging (NODDI), have elucidated distinct patterns of white matter degeneration in AD by providing insights into neurite complexity and orientation dispersion (Veale et al., 2021). These metrics reveal that neurodegenerative processes, characterized by reduced neurite density and altered fiber organization, predominantly affect key regions such as the mesial and lateral temporal lobes (Sone et al., 2020). The diffusion characteristics and structural connectomics of specific regions can provide information for early auxiliary identification of AD (Chen et al., 2023). Khatri and Kwon (2022) have combined sMRI and resting-state functional MRI (rs-fMRI) for efficient biomarker diagnosis and classification of AD, crucial for accurate diagnosis at the initial stages. MRS has shown reduced *N*-acetylaspartate/creatine ratios in the anterior cingulate region, indicating neuronal dysfunction even before structural changes become apparent (Yeh et al., 2018).

While less commonly used than PET or MRI, SPECT offers the ability to use various tracers to assess changes in cerebral perfusion and neurotransmitter dynamics in AD (Herholz, 2011; Sala et al., 2021). For example, SPECT can show changes in cerebral blood flow and neuronal metabolism (Herholz, 2011; Marcolini et al., 2022), as well as their relationships with mood disorders, stress responses, antioxidants, etc. (David et al., 2008; Depboylu et al., 2013). Jeong et al. (2022) used SPECT scanning to investigate the association between regional cerebral blood flow in early AD and neuropsychiatric symptom domains, finding that scores in all neuropsychiatric symptom domains showed correlations with differences in cerebral perfusion. Moreover, SPECT can reveal significant reductions in dopamine receptors in areas such as the basal ganglia and frontal lobe in AD patients, related to neuronal functional impairment (Bajaj et al., 2013) (Table 1).

#### 2.2.3 Therapeutic monitoring and disease progression

The ability to monitor treatment response and track disease progression is crucial for both clinical trials and patient care. Neuroimaging provides objective measures for these assessments:

Therapeutic response monitoring: For example, a study using MRI technology found that BACE inhibitors can cause rapid, regional, and non-progressive reductions in brain volume in AD patients (Sur et al., 2020). Specifically, volumetric MRI analyses indicated a significant increase in brain volume loss associated with verubecestat treatment, particularly in amyloid-rich regions, with the most pronounced hippocampal volume reduction occurring within the first 13 weeks, although no further loss was observed through 78 weeks and without corresponding cognitive decline (Sur et al., 2020). Similarly, another study using PET technology found that plasma exchange could enhance brain metabolism and perfusion in AD patients, especially in cognitively relevant areas such as the temporal and parietal lobes (Cuberas-Borrós et al., 2022). Therefore, neuroimaging techniques can assess not only the effects of pharmacological treatments in AD but also the outcomes of non-pharmacological therapies.

Disease progression and predictive modeling: A study by Li et al. (2018) utilizing MRI and PET technologies found that prognostic models based on multiple longitudinal measurements and time-to-event data could accurately predict cognitive abilities and mortality risks in AD patients. This finding suggests that neuroimaging techniques can provide critical references for the personalized management and intervention of AD patients. Building on this, another study using MRI technology discovered that a model based on deep recurrent neural networks could effectively predict treatment responses and outcomes in AD patients (Jung et al., 2021). Thus, neuroimaging techniques also offer powerful tools and methods for the personalized treatment and evaluation of AD patients, enhancing the ability to tailor interventions to individual needs and monitor their efficacy over time.

### 2.3 Current challenges and future directions

While neuroimaging has revolutionized AD research and clinical practice, several significant challenges remain:

Specificity and differential diagnosis: although neuroimaging can observe abnormal changes in brain structure and function in AD patients, these changes are not entirely specific and may overlap with other neurodegenerative diseases such as Parkinson’s Disease (Carey et al., 2021) and Huntington’s Disease (Estevez-Fraga et al., 2020) or even the normal aging process (Schilling et al., 2022; Blinkouskaya et al., 2021). Consequently, relying solely on neuroimaging techniques is insufficient for accurate diagnosis of AD. It necessitates integration with other clinical assessment indicators, such as biological markers, to enhance diagnostic precision (Graff-Radford et al., 2021).

Technical limitations: current imaging technologies face several modality-specific challenges. While PET imaging provides valuable molecular insights, its widespread application is constrained by high operational costs, limited accessibility, radiation exposure concerns, and the inherent challenge of short tracer half-lives (Berg and Cherry, 2018). Similarly, SPECT imaging, though useful for assessing cerebral perfusion, is hampered by its relatively lower resolution, challenges in image quality, and difficulties in achieving precise quantitative analysis (Livieratos, 2015). The resolution of commonly used neuroimaging techniques like MRI and EEG is still limited in terms of observing the minute structural and functional changes characteristic of early AD (Kim et al., 2022). These techniques cannot directly detect the neural origins of brain volume or thickness loss, nor distinguish whether the loss is due to cell death or the loss of dendrites and synapses (Márquez and Yassa, 2019). Additionally, the need for processing and analyzing large volumes of data poses a challenge in terms of the accuracy and stability of data handling and statistical analysis, which demands high technical proficiency from researchers (Qiu et al., 2020).

Practical implementation barriers: the cost of neuroimaging technology poses a significant challenge. High-resolution brain imaging equipment and the training of specialized personnel require substantial investments, making neuroimaging techniques less accessible in regions with limited resources and medical facilities.

Key priorities include improving the resolution and accuracy of these technologies, reducing costs, and facilitating broader application of neuroimaging techniques in both AD research and clinical practice.

## 3 Gene therapy in AD research

Gene therapy represents a promising therapeutic approach for AD, operating through the delivery of target genes into the cells of a patient via a transduction vector, enabling the production of required proteins or the correction of abnormal gene expression (Brody, 2018), ultimately achieving stable expression of the target genes in the patient’s body. Currently, the main vectors used in AD gene therapy include adeno-associated virus (AAV), lentivirus, and non-viral vectors, each with distinct advantages in terms of targeting efficiency, safety profile, and expression duration (Mendell et al., 2021).

### 3.1 Therapeutic applications in AD

#### 3.1.1 Targeting genetic risk factors

The genetic factors in AD include pathogenic genes and risk genes, which can promote or inhibit the development of AD by affecting the metabolism of Aβ or tau proteins, or influencing pathways such as immune responses, inflammatory responses, and oxidative stress (Scheltens et al., 2021; Jansen et al., 2019). High-throughput sequencing studies have revealed that early-onset AD is primarily associated with mutations in APP, PSEN1, and PSEN2, while late-onset AD involves complex interactions among multiple risk genes (Bertram and Tanzi, 2012). Recent genome-wide association studies (GWAS) and exome sequencing have identified multiple genetic variants associated with AD risk, such as rare variants in genes like *NOTCH3*, *TREM2*, *SORL1*, *ABCA7*, *ATP8B4*, and *ABCA1* (Khani et al., 2022; Bellenguez et al., 2022; Holstege et al., 2022). Gene therapy could target these genetic variants using gene editing techniques like the CRISPR/Cas9 system or gene transfer methods to correct or alter the function of these risk genes, thus reducing the risk of developing AD (Bhardwaj et al., 2022; Thompson, 2024). For example, genome editing of the APP gene’s 3’-UTR in a humanized knock-in mouse model led to reduced Aβ pathology, highlighting the efficacy of CRISPR/Cas9 in mitigating Alzheimer’s Disease through protective mutations (Nagata et al., 2018). One study demonstrated that the CRISPR/Cas9 system could knock out the Swedish APP mutation in fibroblasts derived from patients, resulting in a 39% reduction in Aβ levels (György et al., 2018). The APOE gene, particularly the APOE4 allele, is the strongest genetic risk factor for sporadic AD (Zhao et al., 2020) and serves as a significant biomarker for disease susceptibility (Farrer et al., 1997), making it an important target for gene therapy in AD. Lin et al. (2018) used the CRISPR/Cas9 system in iPSC-derived organoids to convert APOE4 to APOE3, which alleviated multiple AD-related pathologies. Hudry et al. (2013) introduced APOE2 into AD model mice using AAV, reducing the accumulation of A*β* deposits and suggesting that gene transfer to reduce APOE4 or increase APOE2 could help inhibit the progression of AD.

Moreover, gene therapy can also target the expression of specific molecular targets. For example, a therapy strategy based on AAV-mediated knockdown of the CD33 gene successfully reduced the Aβ plaque burden in APP/PS2 mice and significantly lowered levels of the chemokine Ccl33 and the pro-inflammatory factor TNF-*α* (Griciuc et al., 2020). Wang et al. (2016) found that intramuscular delivery of AAV-p75ECD increased the levels of p75ECD in the blood, significantly improving the behavioral phenotype of APP/PS1 transgenic mice, reducing brain amyloid burden, decreasing tau hyperphosphorylation, and attenuating neuroinflammation. Another study found that inducing the AD-like phenotype in normal mice via MST1, and knocking down or chemically inactivating MST1 significantly improved cognitive deficits and neuronal apoptosis in 7-month-old 5xFAD mice (Wang et al., 2022) (Table 2).

**Table 2:** Applications of genetic interventions in Alzheimer’s Disease treatment.

Gene/target	Application	Mechanism/outcome	References
APP	Gene editing	Reduces Aβ pathology; mitigates Alzheimer’s Disease through protective mutations	Nagata et al. (2018) and György et al. (2018)
APOE	Gene therapy	Conversion of APOE4 to APOE3 alleviates AD-related pathologies	Lin et al. (2018) and Hudry et al. (2013)
CD33	AAV-mediated knockdown	Reduces Aβ plaque burden and pro-inflammatory factors; improves cognitive function	Griciuc et al. (2020) and Griciuc et al. (2019)
BACE1	Gene silencing	Decreases amyloid production and neurodegeneration; improves behavioral deficits	Park et al. (2019) and Singer et al. (2005)
NGF	Neuroprotective therapy	Promotes neuronal survival and reduces degeneration; may improve cognitive function	Rafii et al. (2018)
BDNF	Gene delivery	Enhances synaptic plasticity; reduces neuronal loss and synaptic degeneration	Jiao et al. (2016) and Arora et al. (2022)
TREM2	Gene knockout	Reduces microglial activation and neurodegenerative changes	Leyns et al. (2017)
IL-4, IL-10, TGF-β	Plasmid delivery	Modulates inflammatory responses; improves spatial memory performance in AD models	Yoo (2022)
MST1	Knockdown	Improves cognitive deficits and reduces neuronal apoptosis in AD models	Wang et al. (2022)
Rheb	Gene transfer	Activates neurotrophic pathways; enhances neuron survival *in vivo*	Jeon et al. (2020)

#### 3.1.2 Neuroprotection and neural circuit repair in AD

In the context of AD, the concepts of neuroprotection and repair refer to interventions designed to slow down or reverse the process of neuronal damage (Wareham et al., 2022). In a multicenter Phase II trial, AAV2-NGF delivery was tested in AD patients. While AAV2-NGF delivery was well tolerated, it did not affect clinical outcomes or selected AD biomarkers (Rafii et al., 2018). Further analysis revealed that nerve growth factor (NGF) did not directly reach any cholinergic neurons at the injection site, indicating the need for improved vector delivery (Castle et al., 2020). More encouraging results have emerged from preclinical studies. In experiments with mice, Xiao et al. (2023) found that early hippocampal delivery of AAV carrying the gene for Neurotrophic factor-α1/Carboxypeptidase E (NF-α1/CPE) in 3xTg-AD male mice could prevent the later development of cognitive deficits, neurodegeneration, and excessive tau phosphorylation. Additionally, Sun et al. (2019) used the CRISPR-Cas9 system to introduce an early stop codon at the extreme C-terminus of the *APP* gene, inhibiting β-cleavage and Aβ production while promoting α-cleavage, which has neuroprotective effects. Park et al. (2019) used a CRISPR-Cas9 nanoparticle complex that effectively crossed the blood–brain barrier (BBB), entered neurons in adult mice, and produced high-frequency indels at target sites in the BACE1 gene, thereby reducing BACE1 expression and activity. This alleviated Aβ-related pathology and cognitive deficits in two AD mouse models (5XFAD and APP knock-in). Singer et al. (2005) used a lentiviral vector expressing siRNA targeting BACE1 to reduce BACE1 levels, thereby decreasing amyloid production as well as neurodegenerative and behavioral deficits in APP transgenic mice.

Synaptic plasticity is crucial for restoring cognitive function in AD patients (Cuestas Torres and Cardenas, 2020). A key focus has been on neurotrophic factors, particularly Brain-Derived Neurotrophic Factor (BDNF), which plays multiple crucial roles: BDNF plays a key role in promoting nerve growth and maturation, as well as regulating synaptic transmission and plasticity in adulthood (Edelmann et al., 2015; Mizui et al., 2015). However, exogenous BDNF delivery is limited due to its short plasma half-life and limited diffusion across the BBB (Zuccato and Cattaneo, 2009; Pardridge et al., 1994). A study delivered the BDNF gene to the brains of P301L transgenic mice via AAV, resulting in stable expression of BDNF, prevention of neuronal loss, reduction in synaptic degeneration, and fewer neuronal abnormalities (Jiao et al., 2016). Arora et al. (2022) used safer nanoparticles to deliver a plasmid encoding BDNF to the brains of APP/PS1 mice, significantly reducing Aβ and amyloid plaque loads and notably improving synaptic plasticity. NGF is vital for the survival, maintenance, and regeneration of specific neuron populations in the adult brain (Allen et al., 2013). Hohsfield et al. (2013) demonstrated that lentiviral infection could successfully transduce primary rat monocytes and produce effective NGF secretion. Additionally, AAV-2 has been used for NGF delivery, and studies have shown this to be feasible and well-tolerated (Rafii et al., 2014). A study using AAV1-Rheb (S16H) transduced hippocampal neurons induced reactive astrocytes, which produced Ciliary Neurotrophic Factor (CNTF) by activating astrocytic TrkB and upregulating neuronal BDNF and astrocytic CNTF, synergistically aiding the survival of hippocampal neurons *in vivo* (Jeon et al., 2020). Recent research has also shown that AAV11 can effectively retrogradely target projection neurons and enhance astrocytic targeted transduction, making AAV11 a promising tool for mapping and manipulating neural circuits, as well as for gene therapy in neurological and neurodegenerative diseases (Han et al., 2023) (Table 2).

#### 3.1.3 Immunomodulation and neuroinflammation suppression in AD

Recent genetic studies have highlighted the critical role of immune-related genes in AD, opening new avenues for therapeutic intervention. GWAS have identified genetic loci associated with AD, including those related to immune responses and microglia, such as CD33 (Hollingworth et al., 2011; Bertram et al., 2008) and TREM2 (Guerreiro et al., 2013; Jonsson et al., 2013). Griciuc et al. (2019) have demonstrated that knocking out CD33 attenuated A*β* pathology and improved cognitive functions in 5xFAD mice. Additionally, using AAV to deliver artificial microRNAs targeting CD33 into APP/PS1 mice reduced CD33 mRNA levels in brain extracts, as well as TBS-soluble Aβ40 and Aβ42 levels, which are beneficial for mitigating the AD pathological process (Griciuc et al., 2020). Contrary to CD33, in mouse models of tauopathy, knocking out TREM2 reduced microglial activation and improved neurodegenerative changes (Leyns et al., 2017), indicating that further research is needed on targeting TREM2 for AD gene therapy.

Beyond microglial targets, gene therapy can be used to modulate the expression of inflammatory factors such as TNF-*α*, IL-2, and IL-4, effectively treating AD. Griciuc et al. (2020) used AAV to encode artificial microRNAs targeting CD33 in APP/PS1 mice, significantly downregulating pro-inflammatory factors like Tlr4, Ccl2, and TNF-α.

Oxidative stress and chronic neuroinflammation are among the earliest biochemical changes that trigger AD (Prasad, 2017). These early changes present potential therapeutic windows for intervention. Evidence suggests that these early biochemical changes in AD are regulated by small non-coding microRNAs (miR/MiR) (Hernandez-Rapp et al., 2017). Furthermore, most of the upregulated pathogenic genes in AD are under the transcriptional control of pro-inflammatory mediators (Zhao et al., 2016). Studies in patient populations have shown that genetic deficiencies in cytokines like IL-4 and IL-10 increase susceptibility to AD (Li et al., 2014; Su et al., 2016; Babić Leko et al., 2020). Building on this understanding, one study introduced plasmids encoding IL-10, IL-4, TGF-β, or their combination into AβPP mice, resulting in downregulated neuroinflammation and improved spatial memory performance in these mice (Yoo, 2022) (Table 2).

### 3.2 Challenges and future considerations

A primary concern in gene therapy development is the safety and effectiveness. Gene therapy may cause non-specific or non-targeted editing of the genome, leading to genomic instability or oncogenicity (Goswami et al., 2019). The effectiveness of gene therapy may also be affected by factors such as the selection of gene vectors, transduction efficiency, expression level and duration (Cecchin et al., 2023).

Second, the targeting and specificity of gene therapy need to be further improved. Gene therapy needs to precisely deliver genes to damaged neurons or related glial cells to avoid damage to normal cells or tissues (Sudhakar and Richardson, 2019). Additionally, gene therapy must take into account the heterogeneous and multifactorial nature of AD and select appropriate genes and combination strategies to achieve the best therapeutic outcome. Beyond technical challenges, the ethical and social aspects of gene therapy need to be further explored, particularly regarding germline modifications and long-term effects on future generations.

## 4 Application of gut microbiome in AD

### 4.1 Overview of gut microbiota

The gut microbiota constitutes a complex ecosystem together with the host, playing a crucial role in digestion, metabolism, immunity, and neuroendocrine functions. It is also linked to the development of various diseases, including obesity, diabetes, inflammatory bowel diseases, cancer, and neurodegenerative diseases (Lin and Medeiros, 2023; Shi et al., 2023). Recent research has revealed the profound influence of gut microbiota on neurological function through the gut-brain axis, suggesting a novel pathway for understanding and treating AD. Studies have demonstrated that alterations in the gut microbiome can significantly impact brain function and may contribute to neurodegenerative processes through the gut-brain axis (Kesika et al., 2021; Tan et al., 2022).

### 4.2 Gut microbiota in AD pathogenesis

#### 4.2.1 Clinical evidence and epidemiological insights

AD is a multifactorial disease influenced by genetic predispositions and environmental factors throughout a person’s life (Zhang et al., 2021). Emerging evidence has established strong connections between gut dysbiosis and AD development, with multiple pathways linking intestinal health to cognitive function. Studies have shown that dysbiosis can lead to is associated with conditions that increase AD risk, including type 2 diabetes (Ma et al., 2019; Yang et al., 2021), cardiovascular diseases (Rahman et al., 2022; Tang et al., 2019), and hyperhomocysteinemia (Rosario et al., 2021). Notably, research has demonstrated that dietary interventions aimed at correcting gut dysbiosis can prevent Alzheimer’s Disease, indicating that modulation of the gut microbiota can improve AD symptoms (van den Brink et al., 2019; Nagpal et al., 2019). Recent molecular analyses have provided compelling evidence for these connections. Liu et al. (2019) found that the AD patients show distinct gut microbiota compositions that correlated directly with cognitive impairments. This finding has been further supported by 16S ribosomal RNA gene sequencing, which identified similar gut microbiota profiles between AD and MCI patients, profiles that differ significantly from those of healthy individuals (Li et al., 2019). Adding to this evidence, researchers have found that changes in gut microbiota of AD patients linked to their peripheral inflammatory status (Cattaneo et al., 2017), while Haran et al. (2019) used metagenomic sequencing to identify an increase in pro-inflammatory bacteria in the fecal microbiota of AD patients.

#### 4.2.2 Molecular mechanisms and biomarker correlations

Firstly, the gut microbiota can influence the regulation of the immune system, thereby altering the interactions between the immune and nervous systems. The gut microbiota can produce metabolites with pro-inflammatory or anti-inflammatory properties, such as short-chain fatty acids (SCFAs), lipopolysaccharides (LPS), and amino acids (Alkhalaf and Ryan, 2015). These metabolites can enter the brain through the BBB or the vagus nerve, affecting the activity of neurons and glial cells, thus inducing or inhibiting the occurrence of neuroinflammation (Chen et al., 2022; Xu et al., 2023). In the case of microbial dysbiosis, the expression of trigger receptors (TREM-1/2) on bone marrow cells has been described as linking the inflammation process between the gut and neurodegenerative diseases through the microbiota-gut-brain axis (Natale et al., 2019).

Secondly, the gut microbiota can directly or indirectly affect the production and clearance of Aβ. On one hand, the gut microbiota can regulate the synthesis and metabolism of bile acids, influencing cholesterol levels in the liver, and subsequently in the brain (Wahlström et al., 2016). Cholesterol not only regulates the synthesis of Aβ but also controls the interaction between Aβ and neuronal cell membranes. Therefore, an increase in cholesterol levels can promote the production and accumulation of Aβ in the brain (Lockhart and Klimov, 2017). On the other hand, the gut microbiota can produce metabolites with antioxidant, anticoagulant, and blood pressure-regulating effects, such as SCFAs, vitamin K, and hydrogen sulfide, which can affect the function and permeability of cerebral blood vessels, thus influencing the clearance of Aβ (Koszewicz et al., 2021). Moreover, when the gut microbiota is disrupted, pathogenic microbes may replace normal microbes and break through the compromised barriers (Olsen and Yamazaki, 2019), ultimately entering the brain tissue, inducing inflammation and affecting the pathological process of AD (Dando et al., 2014).

What’s more, the gut microbiota can produce or consume precursors or antagonists of neurotransmitters, such as tryptophan, tyrosine, gamma-aminobutyric acid (GABA), dopamine, and serotonin (5-HT) (Collins et al., 2012). These neurotransmitters can enter the brain through the BBB or the vagus nerve, influencing the excitability or inhibition of neurons, thus affecting cognitive functions such as memory, learning, and mood, ultimately leading to cognitive impairment (De-Paula et al., 2018).

Finally, oxidative stress is one of the important causes of AD pathology progression (Bai et al., 2022; Plascencia-Villa and Perry, 2021). Gut bacteria such as bifidobacteria and lactobacilli convert nitrates and nitrites into nitric oxide (NO), increasing the release of NO from host epithelial cells (Oleskin and Shenderov, 2016). Streptococci and bacilli can also produce NO from l-arginine using nitric oxide synthase (Tiso and Schechter, 2015). Furthermore, pathogens such as *Salmonella typhi*, *Escherichia coli*, and Mycobacterium can produce hydrogen sulfide from sulfur-containing amino acids (such as cysteine) in the gastrointestinal tract. High concentrations of hydrogen sulfide can inhibit cyclooxygenase activity, thereby altering glycolytic metabolism, reducing mitochondrial oxygen consumption, decreasing ATP production, and overexpressing pro-inflammatory effects (Leschelle et al., 2005; Beaumont et al., 2016). Thus, the gut microbiota can promote the development of AD directly through oxidative stress or indirectly through promoting neuroinflammation (Bhatt et al., 2020; Łuc et al., 2021).

Beyond these molecular mechanisms, recent studies have revealed important correlations between gut microbiota and AD biomarkers. Gut microbial communities are also closely related to biomarkers of AD. Liu et al. (2019) found that enriched Enterobacteriaceae could be used as markers of AD. Meanwhile, Lu et al. (2024) reported that specific metabolites of intestinal flora, such as indole lactic acid, indole-4-acetaldehyde, and l-proline, could be used as early warning markers of MCI due to AD, and Marizzoni et al. (2020) showed that the metabolites of intestinal microbial communities, LPS and SCFA, were associated with amyloid load and brain amyloid deposition in AD patients. Additionally, biomarkers in cerebrospinal fluid, such as tau protein and Aβ42, are key elements in the pathophysiology of AD (Blennow and Zetterberg, 2018).

### 4.3 The potential of gut microbiota regulation in AD research

While multiple studies have confirmed the association between gut microbiota and AD, three main therapeutic approaches have shown promise in targeting this connection.

Firstly, some studies suggest that probiotics and prebiotics can improve cognitive functions and neuroinflammation in AD patients (Kim et al., 2021). Many probiotics have been used in animal studies and AD models. For instance, in rats, administration of Bifidobacterium and Lactobacillus has shown positive effects on AD treatment, improving memory, learning deficits, and oxidative stress (Azm et al., 2018). In AD mouse models, *Bifidobacterium breve* strain A1 has been shown to block Aβ-induced cognitive dysfunction and inhibit gene expression changes in the hippocampus induced by Aβ (Kobayashi et al., 2017). Additionally, a clinical trial found that administering a probiotic formulation containing Lactobacillus and Bifidobacterium to AD patients could lower serum C-reactive protein levels and improve scores on the Mini-Mental State Examination (Akbari et al., 2016). These findings from both animal and human studies highlight the therapeutic potential of probiotics, though more clinical trials are needed.

Secondly, Fecal Microbiota Transplantation (FMT). FMT involves transferring the fecal microbiota from a healthy individual to the gut of a recipient to restore intestinal microbial balance (Gupta and Khanna, 2017). For example, Sun et al. (2019) found that FMT treatment could improve cognitive deficits and reduce Aβ deposition in the brains of APP/PS1 mice. Kim et al. (2020) discovered that cognitive deficits caused by Aβ and NFTs deposition could be improved through FMT from healthy mouse donors. While these preclinical results are promising, human studies are still limited. These results suggest that FMT might serve as a novel therapeutic approach by modulating the gut microbiota to influence the progression of AD, but more research is needed to verify its effects, mechanisms, and applications in humans. Moreover, since fecal microbiota transplantation is an invasive method, other less invasive approaches such as dietary intervention strategies should be tried first.

The role of antibiotics in AD treatment remains controversial, with studies showing both beneficial and detrimental effects. Antibiotic interference with the gut microbiota could disrupt the balance of the microbiota-gut-brain axis, thus affecting the occurrence and progression of AD. Some studies have found that the use of antibiotics can improve the symptoms and pathology of AD, possibly through mechanisms such as reducing gut and systemic inflammation, reducing the production and deposition of Aβ, increasing the expression of neuroprotective factors, and improving cognitive functions. For example, Minter et al. (2016) found that long-term broad-spectrum antibiotic treatment induced changes in the composition and diversity of the gut microbiota, which reduced Aβ plaque deposition in APP/PS1 mice. Also, multiple studies have shown that rifampicin exhibits strong brain-protective effects in preclinical models of AD, reducing levels of Aβ in the brain and decreasing inflammatory factors (Yulug et al., 2018). However, some studies have found that the use of antibiotics can exacerbate the symptoms and pathology of AD, possibly through mechanisms such as disrupting the diversity and stability of the gut microbiota, lowering levels of beneficial bacteria and metabolites, increasing oxidative stress in the gut and brain, and impairing cognitive functions. For instance, antibiotics like streptomycin have been used to induce sporadic forms of AD in animal models and affect learning and memory performance (Cattaneo et al., 2017; Ravelli et al., 2017). Wang et al. (2015) found that administering ampicillin to rats could increase serum corticosterone, causing anxiety-like behaviors and spatial memory impairments, potentially leading to the exacerbation of AD. The discrepancies in these study results could be related to factors such as the type, dosage, timing of antibiotic use, animal models, evaluation indicators, as well as individual differences and the complexity of the gut microbiota. Therefore, the rational and moderate use of antibiotics, maintaining the balance and health of the gut microbiota, is of significant importance for the prevention and treatment of AD.

In conclusion, the regulation of the gut microbiota holds great potential in AD research, but there are still many challenges, such as the causal relationship between the gut microbiota and AD, the optimal timing and methods for modulating the gut microbiota, and the individual differences and side effects of gut microbiota regulation. Thus, more basic and clinical research is needed to further explore the mechanisms by which the gut microbiota functions in AD, aiming to develop more effective and safer methods of gut microbiota regulation (Table 3).

**Table 3:** Bacterial dysbiosis and antibiotic influence in Alzheimer’s Disease pathogenesis.

Category	Bacteria	Role in AD	References
Pathogenic bacteria	Enterobacteriaceae	Enriched in AD patients, correlated with cognitive impairments	Liu et al. (2019)
	Pro-inflammatory bacteria	Increased in fecal microbiota of AD patients	Haran et al. (2019)
	Bifidobacteria and Lactobacilli	Produce NO and promote oxidative stress	Oleskin and Shenderov (2016)
	*Salmonella typhi*, *Escherichia coli*, and Mycobacterium	Produce hydrogen sulfide, promoting neuroinflammation	Leschelle et al. (2005) and Beaumont et al. (2016)
Beneficial bacteria	Bifidobacterium and Lactobacillus	Can treat AD and improve memory, learning deficiencies, and oxidative stress; Reduces serum C-reactive protein levels and improves scores on the MMSE	Azm et al. (2018) and Akbari et al. (2016)
	*Bifidobacterium breve* strain A1	Can block Aβ-induced cognitive dysfunction and inhibit Aβ-induced changes in hippocampal gene expression	Kobayashi et al. (2017)
Antibiotics	Rifampicin	Exhibits brain-protective effects, reducing Aβ levels	Yulug et al. (2018)
	Broad-spectrum antibiotics	Induced changes in gut microbiota, reducing Aβ plaque deposition	Minter et al. (2016)
	Streptomycin	Affects learning and memory performance and induces a sporadic form of AD in animal models	Cattaneo et al. (2017) and Ravelli et al. (2017)
	Ampicillin	Can increase serum corticosterone, causing cognitive impairment	Wang et al. (2015)

## 5 Strategies for combining neuroimaging techniques, gene therapy, and gut microbiome

### 5.1 Integration principles of neuroimaging techniques, gene therapy, and gut microbiota

The integration of neuroimaging techniques, gene therapy, and gut microbiota offers a novel perspective for studying AD. This integration is based on the principle that neuroimaging techniques allow for the direct monitoring of changes in brain structure and function, gene therapy enables molecular-level regulation and repair of neural damage, and the gut microbiota influences brain health through the gut-brain axis.

Furthermore, advancements in neuroimaging technologies enable unprecedented resolution and dimensionality in observing brain structure and function, such as with PET (Tripathi and Murray, 2022), DTI (Chen et al., 2023), and multimodal MRI (Houria et al., 2022). These technologies allow researchers to directly monitor the specific impacts of gene therapy on brain structure and function and achieve precise targeting of vectors. For example, Ren et al. (2016) utilized translatable MRI to verify the expression of hG-CSF cDNA in living brains, representing a significant non-invasive method for monitoring exogenous gene expression in experimental gene therapy for AD. Convection-enhanced delivery (CED) uses a pressure gradient to create a large infusion of fluid in the interstitial space, enhancing the distribution of large and small molecules in the brain and achieving drug concentrations several orders of magnitude higher than systemic levels (Bobo et al., 1994). Its development allows for efficient, direct, and controlled distribution of viral vector particles throughout the brain. Moreover, real-time MRI-guided CED (iMRI-CED), aimed at monitoring infusion with MRI contrast agents mixed with therapeutic drugs, represents an optimized approach over traditional CED (Varenika et al., 2008; Richardson et al., 2011; Richardson et al., 2011). One study successfully infused AAV2-BDNF into the entorhinal cortex of non-human primates under MRI guidance, achieving safe and precise targeting and distribution of BDNF in the entorhinal cortex and hippocampus (Nagahara et al., 2018). Furthermore, neuroimaging technologies can monitor how changes in the gut microbiota affect brain structure and function. An *ex-vivo* DTI study in rats showed that changes in brain structure were related to diet-dependent changes in the gut microbiota, particularly white matter integrity (Ong et al., 2018). Janik et al. (2016) using MRS and MRI in BALB/c mice, demonstrated that oral administration of *Lactobacillus reuteri* promoted increases in brain GABA, *N*-acetylaspartate, and glutamate. Bagga et al. (2018) found that probiotics could improve memory and alter brain activation patterns. Moreover, a study involving healthy volunteers found functional connectivity changes after a four-week probiotic intervention (Bagga et al., 2019), indicating significant correlations between human gut microbiota characteristics and brain microstructure, intrinsic neural activity, brain functional connectivity, and cognitive and emotional functions. Well-designed longitudinal studies, including assessments of gut microbiota structure and microbial metabolomics, along with neuroimaging and behavioral tests, are needed to establish directionality and causality (Liu et al., 2019).

In particular, recent studies combining neuroimaging with genetic research in AD have identified new targets. Zhao et al. (2022) developed a comprehensive Bayesian genetic power analysis to jointly estimate the heritability of high-dimensional neuroimaging features. Through extensive simulations, they applied this method to two large imaging genetics datasets: the Alzheimer’s Disease Neuroimaging Initiative and the UK Biobank, yielding biologically meaningful results. Ren et al. (2023) assessed the functional connectivity disruptions of the nucleus basalis of Meynert (NbM) in healthy controls and MCI patients using resting-state fMRI data from the ADNI2/GO phase. They explored the transcriptional correlates of NbM connectivity disruptions using public post-mortem whole-brain gene expression datasets from the Allen Human Brain Atlas (AHBA) and the Mount Sinai Brain Bank (MSBB). The results revealed the transcriptional vulnerability of NbM connectivity disruptions and their key role in explaining preclinical Aβ changes and the age of onset in MCI, providing new insights into early AD pathology and encouraging further gene therapy targeting NbM (Ren et al., 2023).

### 5.2 Conceptualization of integrating neuroimaging techniques, gene therapy, and gut microbiota for treating AD

As a foundation, it is essential to establish the causal relationships between gut microbiota dysbiosis and the pathogenesis of AD. Using gene-editing technologies like CRISPR-Cas9, we could develop various transgenic mouse models by knocking out or inserting genes associated with gut microbiota, such as receptors for short-chain fatty acids (FFAR), bile acids (FXR), and neuropeptide Y (NPYR). Through these models, we can assess the resulting alterations in gut microbiota composition, brain Aβ deposition, neuronal integrity, inflammatory markers, and cognitive performance. Such studies will provide invaluable insights into the molecular pathways and regulatory mechanisms connecting gut microbiota to AD pathology.

Additionally, to complement these animal studies, we propose longitudinal cohort studies in humans. These studies should focus on collecting fecal samples from both healthy controls and AD patients. Following sample collection, we can employ 16S rRNA gene sequencing alongside metabolomics to evaluate changes in gut microbiota diversity, enterotypes, and metabolite profiles over time. This longitudinal data will help to establish correlations with clinical phenotypes and biomarker levels indicative of AD, thus laying the groundwork for future therapeutic interventions.

Simultaneously, we must prioritize the development of sophisticated neuroimaging technologies capable of capturing dynamic changes in brain structure, function, and molecular activities, as well as gut microbiota activity. To address the current limitations of resolution and data integration, we propose the implementation of a multimodal fusion approach. This would involve integrating advanced imaging modalities (such as high-resolution MRI, PET, SPECT, fMRI, and MRS) into a unified analytical framework. By utilizing state-of-the-art computational techniques, we can create a comprehensive model that accurately reflects the interactions between the brain and gut microbiota. This model would facilitate the extraction of relevant features and biomarkers through machine learning algorithms, which could be trained on datasets derived from both animal models and human cohorts.

Moreover, incorporating animal research into the development of these imaging techniques will allow for the validation and refinement of our methodologies in controlled settings. For example, we can use mice to evaluate the impact of specific gut microbiota alterations on neuroimaging outcomes, thereby optimizing our approach for human studies. This iterative process will enhance the reliability of the neuroimaging data we obtain, ultimately informing clinical applications.

In tandem with these investigative efforts, a focused exploration of gut microbiota-based therapeutic strategies is essential. We can leverage gene therapy to introduce beneficial genes (such as those encoding NGF or IL-10) into targeted gut microbes like Lactobacillus or Bifidobacterium. These genetically modified organisms can then be administered to AD patients via oral or enema delivery. By colonizing the gut, these microbes would secrete neuroprotective proteins that traverse the gut-brain axis, promoting neurorepair and exerting anti-inflammatory and antioxidant effects directly in the brain. Additionally, we should explore the feasibility of combining these interventions with existing AD therapies, assessing their synergistic potential through controlled clinical trials.

**Figure 1:**
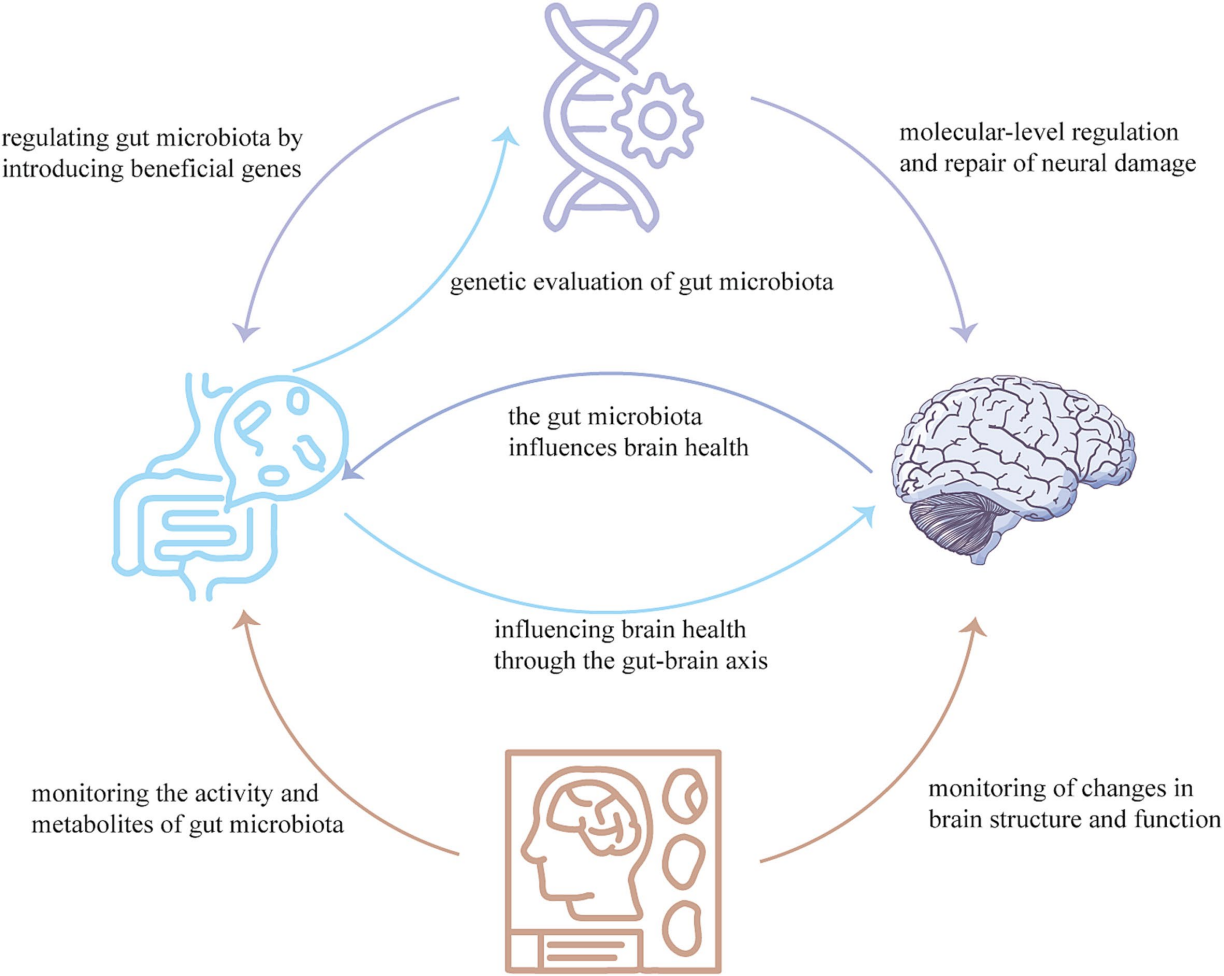
Neuroimaging techniques can monitor the activity and metabolites of the gut microbiota, as well as changes in brain structure and function. Gene therapy can regulate the gut microbiota by producing beneficial genes, and can also regulate and repair neural damage at the molecular-level. The gut microbiome can influence brain health through the gut-brain axis and can also evaluate the effects of gene therapy. These three technologies combined with each other, complement each other, can provide a new means for the diagnosis, treatment, prognosis and monitoring of AD.

In conclusion, through the integration of comprehensive animal studies, advanced human cohort analyses, and cutting-edge neuroimaging technologies, we can elucidate the complex interactions between gut microbiota and AD. The development of targeted therapeutic strategies utilizing gene therapy and modified gut microbes offers a promising avenue for improving AD symptoms and underlying pathology, potentially transforming the management of this debilitating condition (Figure 1).

## 6 Conclusion

In this review, we have highlighted the significant advancements in neuroimaging techniques, gene therapy, and gut microbiota research in the context of Alzheimer’s Disease (AD). These fields not only interconnect but also offer distinct mechanisms that could enhance early diagnosis, improve understanding of disease pathology, and inform novel therapeutic strategies. We propose a conceptual framework that emphasizes the integration of these approaches to foster innovation in AD research. This framework highlights the potential for synergistic effects that could lead to breakthroughs in diagnostic and therapeutic tools, ultimately advancing prevention and intervention strategies. Despite the progress made, several challenges remain. The effectiveness of neuroimaging relies on improvements in resolution and specificity, while gene therapy necessitates enhanced safety and targeted delivery methods. Furthermore, the complex role of gut microbiota in AD pathology requires more thorough investigation to elucidate causal relationships. We advocate for interdisciplinary collaboration to integrate insights and technologies across these fields. Such collaboration is essential to address the current limitations and to harness the full potential of these approaches, paving the way for transformative advances in AD research and ultimately improving patient outcomes.

## References

[ref1] Akbari E. Asemi Z. Daneshvar Kakhaki R. Bahmani F. Kouchaki E. Tamtaji O. R.. (2016). Effect of probiotic supplementation on cognitive function and metabolic status in Alzheimer’s disease: a randomized, double-blind and controlled trial. Front. Aging Neurosci. 8:256. doi: 10.3389/fnagi.2016.00256, PMID: 27891089 PMC5105117

[ref2] Albert M. S. DeKosky S. T. Dickson D. Dubois B. Feldman H. H. Fox N. C.. (2011). The diagnosis of mild cognitive impairment due to Alzheimer’s disease: recommendations from the National Institute on Aging-Alzheimer’s Association workgroups on diagnostic guidelines for Alzheimer’s disease. Alzheimers Dement. 7, 270–279. doi: 10.1016/j.jalz.2011.03.008, PMID: 21514249 PMC3312027

[ref3] Alkhalaf L. M. Ryan K. S. (2015). Biosynthetic manipulation of tryptophan in bacteria: pathways and mechanisms. Chem. Biol. 22, 317–328. doi: 10.1016/j.chembiol.2015.02.005, PMID: 25794436

[ref4] Allen S. J. Watson J. J. Shoemark D. K. Barua N. U. Patel N. K. (2013). GDNF, NGF and BDNF as therapeutic options for neurodegeneration. Pharmacol. Ther. 138, 155–175. doi: 10.1016/j.pharmthera.2013.01.004 23348013

[ref5] Arbabyazd L. Petkoski S. Breakspear M. Solodkin A. Battaglia D. Jirsa V. (2023). State-switching and high-order spatiotemporal organization of dynamic functional connectivity are disrupted by Alzheimer’s disease. Network Neurosci 7, 1420–1451. doi: 10.1162/netn_a_00332, PMID: 38144688 PMC10727776

[ref6] Arora S. Kanekiyo T. Singh J. (2022). Functionalized nanoparticles for brain targeted BDNF gene therapy to rescue Alzheimer’s disease pathology in transgenic mouse model. Int. J. Biol. Macromol. 208, 901–911. doi: 10.1016/j.ijbiomac.2022.03.203, PMID: 35378156

[ref7] Ashrafian H. Zadeh E. H. Khan R. H. (2021). Review on Alzheimer’s disease: inhibition of amyloid beta and tau tangle formation. Int. J. Biol. Macromol. 167, 382–394. doi: 10.1016/j.ijbiomac.2020.11.192 33278431

[ref8] Atri A. (2019). The Alzheimer’s disease clinical Spectrum: diagnosis and management. Med. Clin. North Am. 103, 263–293. doi: 10.1016/j.mcna.2018.10.009 30704681

[ref9] Azm S. A. N. Djazayeri A. Safa M. Azami K. Ahmadvand B. Sabbaghziarani F.. (2018). Lactobacilli and bifidobacteria ameliorate memory and learning deficits and oxidative stress in β-amyloid (1-42) injected rats. Appl. Physiol. Nutr. Metab. 43, 718–726. doi: 10.1139/apnm-2017-0648, PMID: 29462572

[ref10] Babić Leko M. Nikolac Perković M. Klepac N. Štrac D. Borovečki F. Pivac N.. (2020). IL-1β, IL-6, IL-10, and TNFα single nucleotide polymorphisms in human influence the susceptibility to Alzheimer’s disease pathology. J. Alzheimer’s Dis. 75, 1029–1047. doi: 10.3233/JAD-200056, PMID: 32390629

[ref11] Bagga D. Aigner C. S. Reichert J. L. Cecchetto C. Fischmeister F. P. S. Holzer P.. (2019). Influence of 4-week multi-strain probiotic administration on resting-state functional connectivity in healthy volunteers. Eur. J. Nutr. 58, 1821–1827. doi: 10.1007/s00394-018-1732-z, PMID: 29850990 PMC6647073

[ref12] Bagga D. Reichert J. L. Koschutnig K. Aigner C. S. Holzer P. Koskinen K.. (2018). Probiotics drive gut microbiome triggering emotional brain signatures. Gut Microbes 9, 486–496. doi: 10.1080/19490976.2018.1460015, PMID: 29723105 PMC6287679

[ref13] Bai R. Guo J. Ye X. Y. Xie Y. Xie T. (2022). Oxidative stress: the core pathogenesis and mechanism of Alzheimer’s disease. Ageing Res. Rev. 77:101619. doi: 10.1016/j.arr.2022.101619, PMID: 35395415

[ref14] Bajaj N. Hauser R. A. Grachev I. D. (2013). Clinical utility of dopamine transporter single photon emission CT (DaT-SPECT) with (123I) ioflupane in diagnosis of parkinsonian syndromes. J. Neurol. Neurosurg. Psychiatry 84, 1288–1295. doi: 10.1136/jnnp-2012-304436, PMID: 23486993 PMC3812862

[ref15] Beaumont M. Andriamihaja M. Lan A. Khodorova N. Audebert M. Blouin J.-M.. (2016). Detrimental effects for colonocytes of an increased exposure to luminal hydrogen sulfide: the adaptive response. Free Radic. Biol. Med. 93, 155–164. doi: 10.1016/j.freeradbiomed.2016.01.028, PMID: 26849947

[ref16] Bellenguez C. Küçükali F. Jansen I. E. Kleineidam L. Moreno-Grau S. Amin N.. (2022). New insights into the genetic etiology of Alzheimer’s disease and related dementias. Nat. Genet. 54, 412–436. doi: 10.1038/s41588-022-01024-z, PMID: 35379992 PMC9005347

[ref17] Berg E. Cherry S. R. (2018). Innovations in instrumentation for positron emission tomography. Semin. Nucl. Med. 48, 311–331. doi: 10.1053/j.semnuclmed.2018.02.006, PMID: 29852942 PMC5986096

[ref18] Bertram L. Lange C. Mullin K. Parkinson M. Hsiao M. Hogan M. F.. (2008). Genome-wide association analysis reveals putative Alzheimer’s disease susceptibility loci in addition to APOE. Am. J. Hum. Genet. 83, 623–632. doi: 10.1016/j.ajhg.2008.10.008, PMID: 18976728 PMC2668052

[ref19] Bertram L. Tanzi R. E. (2012). The genetics of Alzheimer’s disease. Prog. Mol. Biol. Transl. Sci. 107, 79–100. doi: 10.1016/B978-0-12-385883-2.00008-4 22482448

[ref20] Bhardwaj S. Kesari K. K. Rachamalla M. Mani S. Ashraf G. M. Jha S. K.. (2022). CRISPR/Cas9 gene editing: new hope for Alzheimer’s disease therapeutics. J. Adv. Res. 40, 207–221. doi: 10.1016/j.jare.2021.07.001, PMID: 36100328 PMC9481950

[ref21] Bhatt S. Nagappa A. N. Patil C. R. (2020). Role of oxidative stress in depression. Drug Discov. Today 25, 1270–1276. doi: 10.1016/j.drudis.2020.05.001 32404275

[ref22] Blamire A. M. (2018). MR approaches in neurodegenerative disorders. Prog. Nucl. Magn. Reson. Spectrosc. 108, 1–16. doi: 10.1016/j.pnmrs.2018.11.001 30538047

[ref23] Blennow K. Zetterberg H. (2018). Biomarkers for Alzheimer’s disease: current status and prospects for the future. J. Intern. Med. 284, 643–663. doi: 10.1111/joim.12816, PMID: 30051512

[ref24] Blinkouskaya Y. Caçoilo A. Gollamudi T. Jalalian S. Weickenmeier J. (2021). Brain aging mechanisms with mechanical manifestations. Mech. Ageing Dev. 200:111575. doi: 10.1016/j.mad.2021.111575, PMID: 34600936 PMC8627478

[ref25] Bobo R. H. Laske D. W. Akbasak A. Morrison P. F. Dedrick R. L. Oldfield E. H. (1994). Convection-enhanced delivery of macromolecules in the brain. Proc. Natl. Acad. Sci. USA 91, 2076–2080. doi: 10.1073/pnas.91.6.2076, PMID: 8134351 PMC43312

[ref26] Brody H. (2018). Gene therapy. Nature 564:S5. doi: 10.1038/d41586-018-07639-9 30542191

[ref27] Canova D. Roatta S. Micieli G. Bosone D. (2012). Cerebral oxygenation and haemodynamic effects induced by nimodipine in healthy subjects. Funct. Neurol. 27, 169–176, PMID: 23402678 PMC3812769

[ref28] Carey G. Görmezoğlu M. de Jong J. J. A. Hofman P. A. M. Backes W. H. Dujardin K.. (2021). Neuroimaging of anxiety in Parkinson’s disease: a systematic review. Mov. Disord 36, 327–339. doi: 10.1002/mds.28404, PMID: 33289195 PMC7984351

[ref29] Castle M. J. Baltanás F. C. Kovacs I. Nagahara A. H. Barba D. Tuszynski M. H. (2020). Postmortem analysis in a clinical trial of AAV2-NGF gene therapy for Alzheimer’s disease identifies a need for improved vector delivery. Hum. Gene Ther. 31, 415–422. doi: 10.1089/hum.2019.367, PMID: 32126838 PMC7194314

[ref30] Cattaneo A. Cattane N. Galluzzi S. Provasi S. Lopizzo N. Festari C.. (2017). Association of brain amyloidosis with pro-inflammatory gut bacterial taxa and peripheral inflammation markers in cognitively impaired elderly. Neurobiol. Aging 49, 60–68. doi: 10.1016/j.neurobiolaging.2016.08.019, PMID: 27776263

[ref31] Cecchin R. Troyer Z. Witwer K. Morris K. V. (2023). Extracellular vesicles: the next generation in gene therapy delivery. Mol. Ther. 31, 1225–1230. doi: 10.1016/j.ymthe.2023.01.021, PMID: 36698310 PMC10188631

[ref32] Chandra A. Valkimadi P. E. Pagano G. Cousins O. Dervenoulas G. Politis M. (2019). Applications of amyloid, tau, and neuroinflammation PET imaging to Alzheimer’s disease and mild cognitive impairment. Hum. Brain Mapp. 40, 5424–5442. doi: 10.1002/hbm.24782, PMID: 31520513 PMC6864887

[ref33] Chaney A. M. Lopez-Picon F. R. Serrière S. Wang R. Bochicchio D. Webb S. D.. (2021). Prodromal neuroinflammatory, cholinergic and metabolite dysfunction detected by PET and MRS in the TgF344-AD transgenic rat model of AD: a collaborative multi-modal study. Theranostics 11, 6644–6667. doi: 10.7150/thno.56059, PMID: 34093845 PMC8171096

[ref34] Chen C. Liao J. Xia Y. Liu X. Jones R. Haran J.. (2022). Gut microbiota regulate Alzheimer’s disease pathologies and cognitive disorders via PUFA-associated neuroinflammation. Gut 71, 2233–2252. doi: 10.1136/gutjnl-2021-326269, PMID: 35017199 PMC10720732

[ref35] Chen X. Steckner M. (2017). Electromagnetic computation and modeling in MRI. Med. Phys. 44, 1186–1203. doi: 10.1002/mp.12103 28079264

[ref36] Chen Y. Wang Y. Song Z. Fan Y. Gao T. Tang X. (2023). Abnormal white matter changes in Alzheimer’s disease based on diffusion tensor imaging: a systematic review. Ageing Res. Rev. 87:101911. doi: 10.1016/j.arr.2023.101911, PMID: 36931328

[ref37] Chételat G. (2018). Multimodal neuroimaging in Alzheimer’s disease: early diagnosis, Physiopathological mechanisms, and impact of lifestyle. J. Alzheimer’s Dis. 64, S199–s211. doi: 10.3233/JAD-179920, PMID: 29504542 PMC6004909

[ref38] Choe Y. M. Byun M. S. Yi D. Lee J. H. Jeon S. Y. Sohn B. K.. (2019). Sleep experiences during different lifetime periods and in vivo Alzheimer pathologies. Alzheimers Res. Ther. 11:79. doi: 10.1186/s13195-019-0536-6, PMID: 31511066 PMC6739958

[ref39] Chouliaras L. Thomas A. Malpetti M. Donaghy P. Kane J. Mak E.. (2022). Differential levels of plasma biomarkers of neurodegeneration in Lewy body dementia, Alzheimer’s disease, frontotemporal dementia and progressive supranuclear palsy. J. Neurol. Neurosurg. Psychiatry 93, 651–658. doi: 10.1136/jnnp-2021-327788, PMID: 35078917 PMC9148982

[ref40] Collins S. M. Surette M. Bercik P. (2012). The interplay between the intestinal microbiota and the brain. Nat. Rev. Microbiol. 10, 735–742. doi: 10.1038/nrmicro2876 23000955

[ref41] Colloby S. J. Field R. H. Wyper D. J. O’Brien J. T. Taylor J. P. (2016). A spatial covariance (123)I-5IA-85380 SPECT study of α4β2 nicotinic receptors in Alzheimer’s disease. Neurobiol. Aging 47, 83–90. doi: 10.1016/j.neurobiolaging.2016.07.017, PMID: 27565302 PMC5082764

[ref42] Companys-Alemany J. Turcu A. L. Vázquez S. Pallàs M. Griñán-Ferré C. (2022). Glial cell reactivity and oxidative stress prevention in Alzheimer’s disease mice model by an optimized NMDA receptor antagonist. Sci. Rep. 12:17908. doi: 10.1038/s41598-022-22963-x, PMID: 36284170 PMC9596444

[ref43] Cuberas-Borrós G. Roca I. Castell-Conesa J. Núñez L. Boada M. López O. L.. (2022). Neuroimaging analyses from a randomized, controlled study to evaluate plasma exchange with albumin replacement in mild-to-moderate Alzheimer’s disease: additional results from the AMBAR study. Eur. J. Nucl. Med. Mol. Imaging 49, 4589–4600. doi: 10.1007/s00259-022-05915-5, PMID: 35867135 PMC9606044

[ref44] Cuestas Torres D. M. Cardenas F. P. (2020). Synaptic plasticity in Alzheimer’s disease and healthy aging. Rev. Neurosci. 31, 245–268. doi: 10.1515/revneuro-2019-0058 32250284

[ref45] Dando S. J. Mackay-Sim A. Norton R. Currie B. J. St John J. A. Ekberg J. A.. (2014). Pathogens penetrating the central nervous system: infection pathways and the cellular and molecular mechanisms of invasion. Clin. Microbiol. Rev. 27, 691–726. doi: 10.1128/CMR.00118-13, PMID: 25278572 PMC4187632

[ref46] Das T. K. Ganesh B. P. (2023). Interlink between the gut microbiota and inflammation in the context of oxidative stress in Alzheimer’s disease progression. Gut Microbes 15:2206504. doi: 10.1080/19490976.2023.2206504, PMID: 37127846 PMC10153019

[ref47] David R. Koulibaly M. Benoit M. Garcia R. Caci H. Darcourt J.. (2008). Striatal dopamine transporter levels correlate with apathy in neurodegenerative diseases a SPECT study with partial volume effect correction. Clin. Neurol. Neurosurg. 110, 19–24. doi: 10.1016/j.clineuro.2007.08.007 17900799

[ref48] De Santi S. de Leon M. J. Rusinek H. Convit A. Tarshish C. Y. Roche A.. (2001). Hippocampal formation glucose metabolism and volume losses in MCI and AD. Neurobiol. Aging 22, 529–539. doi: 10.1016/S0197-4580(01)00230-5, PMID: 11445252

[ref49] De-Paula V. d. J. R. Forlenza A. S. Forlenza O. V. (2018). Relevance of gutmicrobiota in cognition, behaviour and Alzheimer’s disease. Pharmacol. Res. 136, 29–34. doi: 10.1016/j.phrs.2018.07.007, PMID: 30138667

[ref50] Depboylu C. Maurer L. Matusch A. Hermanns G. Windolph A. Béhé M.. (2013). Effect of long-term treatment with pramipexole or levodopa on presynaptic markers assessed by longitudinal [123I]FP-CIT SPECT and histochemistry. NeuroImage 79, 191–200. doi: 10.1016/j.neuroimage.2013.04.076 23631981

[ref51] Dou X. Yao H. Feng F. Wang P. Zhou B. Jin D.. (2020). Characterizing white matter connectivity in Alzheimer’s disease and mild cognitive impairment: An automated fiber quantification analysis with two independent datasets. Cortex 129, 390–405. doi: 10.1016/j.cortex.2020.03.032 32574842

[ref52] Edelmann E. Cepeda-Prado E. Franck M. Lichtenecker P. Brigadski T. Leßmann V. (2015). Theta burst firing recruits BDNF release and signaling in postsynaptic CA1 neurons in spike-timing-dependent LTP. Neuron 86, 1041–1054. doi: 10.1016/j.neuron.2015.04.007, PMID: 25959732

[ref53] El Haj M. Boutoleau-Bretonnière C. Chapelet G. (2023). ChatGPT’s dance with neuropsychological data: a case study in Alzheimer’s disease. Ageing Res. Rev. 92:102117. doi: 10.1016/j.arr.2023.102117, PMID: 37926396

[ref54] Estevez-Fraga C. Scahill R. Rees G. Tabrizi S. J. Gregory S. (2020). Diffusion imaging in Huntington’s disease: comprehensive review. J. Neurol. Neurosurg. Psychiatry 92, 62–69. doi: 10.1136/jnnp-2020-324377, PMID: 33033167 PMC7803908

[ref55] Farrer L. A. Cupples L. A. Haines J. L. Hyman B. Kukull W. A. Mayeux R.. (1997). Effects of age, sex, and ethnicity on the association between apolipoprotein E genotype and Alzheimer disease. A meta-analysis. APOE and Alzheimer Disease Meta Analysis Consortium. JAMA 278, 1349–1356. doi: 10.1001/jama.1997.03550160069041, PMID: 9343467

[ref56] Frisoni G. B. (2001). Structural imaging in the clinical diagnosis of Alzheimer’s disease: problems and tools. J. Neurol. Neurosurg. Psychiatry 70, 711–718. doi: 10.1136/jnnp.70.6.711, PMID: 11384998 PMC1737393

[ref57] Gaubert S. Raimondo F. Houot M. Corsi M. C. Naccache L. Diego Sitt J.. (2019). EEG evidence of compensatory mechanisms in preclinical Alzheimer’s disease. Brain 142, 2096–2112. doi: 10.1093/brain/awz150, PMID: 31211359

[ref58] Goswami R. Subramanian G. Silayeva L. Newkirk I. Doctor D. Chawla K.. (2019). Gene therapy leaves a vicious cycle. Front. Oncol. 9:297. doi: 10.3389/fonc.2019.00297, PMID: 31069169 PMC6491712

[ref59] Graff-Radford J. Yong K. X. X. Apostolova L. G. Bouwman F. H. Carrillo M. Dickerson B. C.. (2021). New insights into atypical Alzheimer’s disease in the era of biomarkers. Lancet Neurol. 20, 222–234. doi: 10.1016/S1474-4422(20)30440-3 33609479 PMC8056394

[ref60] Griciuc A. Federico A. N. Natasan J. Forte A. M. McGinty D. Nguyen H.. (2020). Gene therapy for Alzheimer’s disease targeting CD33 reduces amyloid beta accumulation and neuroinflammation. Hum. Mol. Genet. 29, 2920–2935. doi: 10.1093/hmg/ddaa179, PMID: 32803224 PMC7566501

[ref61] Griciuc A. Patel S. Federico A. N. Choi S. H. Innes B. J. Oram M. K.. (2019). TREM2 acts downstream of CD33 in modulating microglial pathology in Alzheimer’s disease. Neuron 103, 820–35.e7. doi: 10.1016/j.neuron.2019.06.010, PMID: 31301936 PMC6728215

[ref62] Guerreiro R. Wojtas A. Bras J. Carrasquillo M. Rogaeva E. Majounie E.. (2013). TREM2 variants in Alzheimer’s disease. N. Engl. J. Med. 368, 117–127. doi: 10.1056/NEJMoa1211851, PMID: 23150934 PMC3631573

[ref63] Gupta A. Khanna S. (2017). Fecal microbiota transplantation. JAMA 318:1:102. doi: 10.1001/jama.2017.6466 28672320

[ref64] György B. Lööv C. Zaborowski M. P. Takeda S. Kleinstiver B. P. Commins C.. (2018). CRISPR/Cas9 mediated disruption of the Swedish APP allele as a therapeutic approach for early-onset Alzheimer’s disease. Mol. Ther. 11, 429–440. doi: 10.1016/j.omtn.2018.03.007, PMID: 29858078 PMC5992788

[ref65] Hampel H. Teipel S. J. Alexander G. E. Pogarell O. Rapoport S. I. Möller H. J. (2002). In vivo imaging of region and cell type specific neocortical neurodegeneration in Alzheimer’s disease. Perspectives of MRI derived corpus callosum measurement for mapping disease progression and effects of therapy. Evidence from studies with MRI, EEG and PET. J. Neural Transm 109, 837–855. doi: 10.1007/s007020200069 12111472

[ref66] Han Z. Luo N. Ma W. Liu X. Cai Y. Kou J.. (2023). AAV11 enables efficient retrograde targeting of projection neurons and enhances astrocyte-directed transduction. Nat. Commun. 14:3792. doi: 10.1038/s41467-023-39554-7, PMID: 37365155 PMC10293207

[ref67] Haran J. P. Bhattarai S. K. Foley S. E. Dutta P. Ward D. V. Bucci V.. (2019). Alzheimer’s disease microbiome is associated with dysregulation of the anti-inflammatory P-glycoprotein pathway. MBio 10:e00632-19. doi: 10.1128/mBio.00632-19, PMID: 31064831 PMC6509190

[ref68] Herholz K. (2011). Perfusion SPECT and FDG-PET. Int. Psychogeriatr. 23, S25–S31. doi: 10.1017/S1041610211000937 21729421

[ref69] Hernandez-Rapp J. Rainone S. Hébert S. S. (2017). MicroRNAs underlying memory deficits in neurodegenerative disorders. Prog. Neuro-Psychopharmacol. Biol. Psychiatry 73, 79–86. doi: 10.1016/j.pnpbp.2016.04.011 27117821

[ref70] Hohsfield L. A. Geley S. Reindl M. Humpel C. (2013). The generation of NGF-secreting primary rat monocytes: a comparison of different transfer methods. J. Immunol. Methods 391, 112–124. doi: 10.1016/j.jim.2013.02.016, PMID: 23474426 PMC3638233

[ref71] Hollingworth P. Harold D. Sims R. Gerrish A. Lambert J. C. Carrasquillo M. M.. (2011). Common variants at ABCA7, MS4A6A/MS4A4E, EPHA1, CD33 and CD2AP are associated with Alzheimer’s disease. Nat. Genet. 43, 429–435. doi: 10.1038/ng.803, PMID: 21460840 PMC3084173

[ref72] Holstege H. Hulsman M. Charbonnier C. Grenier-Boley B. Quenez O. Grozeva D.. (2022). Exome sequencing identifies rare damaging variants in ATP8B4 and ABCA1 as risk factors for Alzheimer’s disease. Nat. Genet. 54, 1786–1794. doi: 10.1038/s41588-022-01208-7, PMID: 36411364 PMC9729101

[ref73] Houria L. Belkhamsa N. Cherfa A. Cherfa Y. (2022). Multi-modality MRI for Alzheimer’s disease detection using deep learning. Phys. Eng. Sci. Med. 45, 1043–1053. doi: 10.1007/s13246-022-01165-9 36063346

[ref74] Hudry E. Dashkoff J. Roe A. D. Takeda S. Koffie R. M. Hashimoto T.. (2013). Gene transfer of human Apoe isoforms results in differential modulation of amyloid deposition and neurotoxicity in mouse brain. Sci. Transl. Med. 5:7000. doi: 10.1126/scitranslmed.3007000 PMC4334150 24259049

[ref75] Jang J. W. Kim J. Park S. W. Kasani P. H. Kim Y. Kim S.. (2022). Machine learning-based automatic estimation of cortical atrophy using brain computed tomography images. Sci. Rep. 12:14740. doi: 10.1038/s41598-022-18696-6, PMID: 36042322 PMC9427760

[ref76] Janik R. Thomason L. A. M. Stanisz A. M. Forsythe P. Bienenstock J. Stanisz G. J. (2016). Magnetic resonance spectroscopy reveals oral Lactobacillus promotion of increases in brain GABA, N-acetyl aspartate and glutamate. NeuroImage 125, 988–995. doi: 10.1016/j.neuroimage.2015.11.018 26577887

[ref77] Jansen I. E. Savage J. E. Watanabe K. Bryois J. Williams D. M. Steinberg S.. (2019). Genome-wide meta-analysis identifies new loci and functional pathways influencing Alzheimer’s disease risk. Nat. Genet. 51, 404–413. doi: 10.1038/s41588-018-0311-9, PMID: 30617256 PMC6836675

[ref78] Jeon M. T. Moon G. J. Kim S. Choi M. Oh Y. S. Kim D. W.. (2020). Neurotrophic interactions between neurons and astrocytes following AAV1-Rheb(S16H) transduction in the hippocampus in vivo. Br. J. Pharmacol. 177, 668–686. doi: 10.1111/bph.14882, PMID: 31658360 PMC7012949

[ref79] Jeong H. Kang I. Park J. S. Na S. H. Kim S. Yoon S.. (2022). Regional cerebral blood flow correlates of neuropsychiatric symptom domains in early Alzheimer’s disease. Diagnostics 12:1246. doi: 10.3390/diagnostics12051246 35626401 PMC9140211

[ref80] Ji B. Ono M. Yamasaki T. Fujinaga M. Zhang M. R. Seki C.. (2021). Detection of Alzheimer’s disease-related neuroinflammation by a PET ligand selective for glial versus vascular translocator protein. J. Cereb Blood Flow Metab. 41, 2076–2089. doi: 10.1177/0271678X21992457, PMID: 33557690 PMC8327108

[ref81] Jiao S. S. Shen L. L. Zhu C. Bu X. L. Liu Y. H. Liu C. H.. (2016). Brain-derived neurotrophic factor protects against tau-related neurodegeneration of Alzheimer’s disease. Transl. Psychiatry 6:e907. doi: 10.1038/tp.2016.186, PMID: 27701410 PMC5315549

[ref82] Jin Y. Huang C. Daianu M. Zhan L. Dennis E. L. Reid R. I.. (2017). 3D tract-specific local and global analysis of white matter integrity in Alzheimer’s disease. Hum. Brain Mapp. 38, 1191–1207. doi: 10.1002/hbm.23448, PMID: 27883250 PMC5299040

[ref83] Jonsson T. Stefansson H. Steinberg S. Jonsdottir I. Jonsson P. V. Snaedal J.. (2013). Variant of TREM2 associated with the risk of Alzheimer’s disease. N. Engl. J. Med. 368, 107–116. doi: 10.1056/NEJMoa1211103, PMID: 23150908 PMC3677583

[ref84] Josephs K. A. Whitwell J. L. Dickson D. W. Boeve B. F. Knopman D. S. Petersen R. C.. (2008). Voxel-based morphometry in autopsy proven PSP and CBD. Neurobiol. Aging 29, 280–289. doi: 10.1016/j.neurobiolaging.2006.09.019, PMID: 17097770 PMC2702857

[ref85] Jung W. Jun E. Suk H. I. (2021). Deep recurrent model for individualized prediction of Alzheimer’s disease progression. NeuroImage 237:118143. doi: 10.1016/j.neuroimage.2021.118143 33991694

[ref86] Kesika P. Suganthy N. Sivamaruthi B. S. Chaiyasut C. (2021). Role of gut-brain axis, gut microbial composition, and probiotic intervention in Alzheimer’s disease. Life Sci. 264:118627. doi: 10.1016/j.lfs.2020.118627 33169684

[ref87] Khani M. Gibbons E. Bras J. Guerreiro R. (2022). Challenge accepted: uncovering the role of rare genetic variants in Alzheimer’s disease. Mol. Neurodegener. 17:3. doi: 10.1186/s13024-021-00505-9, PMID: 35000612 PMC8744312

[ref88] Khatri U. Kwon G. R. (2022). Alzheimer’s disease diagnosis and biomarker analysis using resting-state functional MRI functional brain network with multi-measures features and hippocampal subfield and amygdala volume of structural MRI. Front. Aging Neurosci. 14:818871. doi: 10.3389/fnagi.2022.818871, PMID: 35707703 PMC9190953

[ref89] Kim C. S. Cha L. Sim M. Jung S. Chun W. Y. Baik H. W.. (2021). Probiotic supplementation improves cognitive function and mood with changes in gut microbiota in community-dwelling older adults: a randomized, double-blind, placebo-controlled, multicenter trial. J. Gerontol. A Biol. Sci. Med. Sci. 76, 32–40. doi: 10.1093/gerona/glaa090, PMID: 32300799 PMC7861012

[ref90] Kim J. Jeong M. Stiles W. R. Choi H. S. (2022). Neuroimaging modalities in Alzheimer’s disease: diagnosis and clinical features. Int. J. Mol. Sci. 23:6079. doi: 10.3390/ijms23116079 35682758 PMC9181385

[ref91] Kim M.-S. Kim Y. Choi H. Kim W. Park S. Lee D.. (2020). Transfer of a healthy microbiota reduces amyloid and tau pathology in an Alzheimer’s disease animal model. Gut 69, 283–294. doi: 10.1136/gutjnl-2018-317431 31471351

[ref92] Kobayashi Y. Sugahara H. Shimada K. Mitsuyama E. Kuhara T. Yasuoka A.. (2017). Therapeutic potential of *Bifidobacterium breve* strain A1 for preventing cognitive impairment in Alzheimer’s disease. Sci. Rep. 7:13510. doi: 10.1038/s41598-017-13368-2, PMID: 29044140 PMC5647431

[ref93] Koszewicz M. Jaroch J. Brzecka A. Ejma M. Budrewicz S. Mikhaleva L. M.. (2021). Dysbiosis is one of the risk factor for stroke and cognitive impairment and potential target for treatment. Pharmacol. Res. 164:105277. doi: 10.1016/j.phrs.2020.105277, PMID: 33166735

[ref94] Leandrou S. Petroudi S. Kyriacou P. A. Reyes-Aldasoro C. C. Pattichis C. S. (2018). Quantitative MRI brain studies in mild cognitive impairment and Alzheimer’s disease: a methodological review. IEEE Rev. Biomed. Eng. 11, 97–111. doi: 10.1109/RBME.2018.2796598, PMID: 29994606

[ref95] Leschelle X. Goubern M. Andriamihaja M. Blottière H. M. Couplan E. M-d-M G.-B.. (2005). Adaptative metabolic response of human colonic epithelial cells to the adverse effects of the luminal compound sulfide. Biochim. Biophys. Acta Gen. Subj. 1725, 201–212. doi: 10.1016/j.bbagen.2005.06.002 15996823

[ref96] Levin F. Ferreira D. Lange C. Dyrba M. Westman E. Buchert R.. (2021). Data-driven FDG-PET subtypes of Alzheimer’s disease-related neurodegeneration. Alzheimers Res. Ther. 13:49. doi: 10.1186/s13195-021-00785-9, PMID: 33608059 PMC7896407

[ref97] Leyns C. E. G. Ulrich J. D. Finn M. B. Stewart F. R. Koscal L. J. Remolina Serrano J.. (2017). TREM2 deficiency attenuates neuroinflammation and protects against neurodegeneration in a mouse model of tauopathy. Proc. Natl. Acad. Sci. USA 114, 11524–11529. doi: 10.1073/pnas.1710311114, PMID: 29073081 PMC5663386

[ref98] Li B. He Y. Ma J. Huang P. Du J. Cao L.. (2019). Mild cognitive impairment has similar alterations as Alzheimer’s disease in gut microbiota. Alzheimers Dement. 15, 1357–1366. doi: 10.1016/j.jalz.2019.07.002, PMID: 31434623

[ref99] Li K. O’Brien R. Lutz M. Luo S. (2018). A prognostic model of Alzheimer’s disease relying on multiple longitudinal measures and time-to-event data. Alzheimers Dement. 14, 644–651. doi: 10.1016/j.jalz.2017.11.004, PMID: 29306668 PMC5938096

[ref100] Li W. Qian X. Teng H. Ding Y. Zhang L. (2014). Association of interleukin-4 genetic polymorphisms with sporadic Alzheimer’s disease in Chinese Han population. Neurosci. Lett. 563, 17–21. doi: 10.1016/j.neulet.2014.01.019, PMID: 24463336

[ref101] Lin D. Medeiros D. M. (2023). The microbiome as a major function of the gastrointestinal tract and its implication in micronutrient metabolism and chronic diseases. Nutr. Res. 112, 30–45. doi: 10.1016/j.nutres.2023.02.007, PMID: 36965327

[ref102] Lin Y. T. Seo J. Gao F. Feldman H. M. Wen H. L. Penney J.. (2018). APOE4 causes widespread molecular and cellular alterations associated with Alzheimer’s disease phenotypes in human iPSC-derived brain cell types. Neuron 98, 1141–54.e7. doi: 10.1016/j.neuron.2018.05.008, PMID: 29861287 PMC6023751

[ref103] Lista S. Vergallo A. Teipel S. J. Lemercier P. Giorgi F. S. Gabelle A.. (2023). Determinants of approved acetylcholinesterase inhibitor response outcomes in Alzheimer’s disease: relevance for precision medicine in neurodegenerative diseases. Ageing Res. Rev. 84:101819. doi: 10.1016/j.arr.2022.101819, PMID: 36526257

[ref104] Liu P. Peng G. Zhang N. Wang B. Luo B. (2019). Crosstalk between the gut microbiota and the brain: An update on neuroimaging findings. Front. Neurol. 10:883. doi: 10.3389/fneur.2019.00883, PMID: 31456743 PMC6700295

[ref105] Liu P. Wu L. Peng G. Han Y. Tang R. Ge J.. (2019). Altered microbiomes distinguish Alzheimer’s disease from amnestic mild cognitive impairment and health in a Chinese cohort. Brain Behav. Immun. 80, 633–643. doi: 10.1016/j.bbi.2019.05.008, PMID: 31063846

[ref106] Livieratos L. (2015). Technical pitfalls and limitations of SPECT/CT. Semin. Nucl. Med. 45, 530–540. doi: 10.1053/j.semnuclmed.2015.06.002, PMID: 26522394

[ref107] Lockhart C. Klimov D. K. (2017). Cholesterol changes the mechanisms of Aβ peptide binding to the DMPC bilayer. J. Chem. Inf. Model. 57, 2554–2565. doi: 10.1021/acs.jcim.7b00431, PMID: 28910085

[ref108] Lu L. Qin L. Zhao X. Liu Z. Qiu X. Yang S.. (2024). Metabolites of intestinal fora can be used as diagnostic and progressive markers for mild cognitive impairment. Front. Cell. Infect. Microbiol. 14:1351523. doi: 10.3389/fcimb.2024.1351523 38404286 PMC10885801

[ref109] Łuc M. Misiak B. Pawłowski M. Stańczykiewicz B. Zabłocka A. Szcześniak D.. (2021). Gut microbiota in dementia. Critical review of novel findings and their potential application. Prog. Neuropsychopharmacol. Biol. Psychiatry 104:110039. doi: 10.1016/j.pnpbp.2020.110039 32687964

[ref110] Ma Q. Li Y. Li P. Wang M. Wang J. Tang Z.. (2019). Research progress in the relationship between type 2 diabetes mellitus and intestinal flora. Biomed. Pharmacother. 117:109138. doi: 10.1016/j.biopha.2019.109138 31247468

[ref111] Marcolini S. Frentz I. Sanchez-Catasus C. A. Mondragon J. D. Feltes P. K. van der Hoorn A.. (2022). Effects of interventions on cerebral perfusion in the Alzheimer’s disease spectrum: a systematic review. Ageing Res. Rev. 79:101661. doi: 10.1016/j.arr.2022.101661 35671869

[ref112] Marizzoni M. Cattaneo A. Mirabelli P. Festari C. Lopizzo N. Nicolosi V.. (2020). Short-chain fatty acids and lipopolysaccharide as mediators between gut Dysbiosis and amyloid pathology in Alzheimer’s disease. J. Alzheimers Dis. 78, 683–697. doi: 10.3233/JAD-200306, PMID: 33074224

[ref113] Márquez F. Yassa M. A. (2019). Neuroimaging biomarkers for Alzheimer’s disease. Mol. Neurodegener. 14:21. doi: 10.1186/s13024-019-0325-5, PMID: 31174557 PMC6555939

[ref114] Mendell J. R. Al-Zaidy S. A. Rodino-Klapac L. R. Goodspeed K. Gray S. J. Kay C. N.. (2021). Current clinical applications of in vivo gene therapy with AAVs. Mol. Ther. 29, 464–488. doi: 10.1016/j.ymthe.2020.12.007, PMID: 33309881 PMC7854298

[ref115] Minter M. R. Zhang C. Leone V. Ringus D. L. Zhang X. Oyler-Castrillo P.. (2016). Antibiotic-induced perturbations in gut microbial diversity influences neuro-inflammation and amyloidosis in a murine model of Alzheimer’s disease. Sci. Rep. 6:30028. doi: 10.1038/srep30028, PMID: 27443609 PMC4956742

[ref116] Mizui T. Ishikawa Y. Kumanogoh H. Lume M. Matsumoto T. Hara T.. (2015). BDNF pro-peptide actions facilitate hippocampal LTD and are altered by the common BDNF polymorphism Val66Met. Proc. Natl. Acad. Sci. USA 112, E3067–E3074. doi: 10.1073/pnas.1422336112, PMID: 26015580 PMC4466729

[ref117] Nagahara A. H. Wilson B. R. Ivasyk I. Kovacs I. Rawalji S. Bringas J. R.. (2018). MR-guided delivery of AAV2-BDNF into the entorhinal cortex of non-human primates. Gene Ther. 25, 104–114. doi: 10.1038/s41434-018-0010-2, PMID: 29535375 PMC5924461

[ref118] Nagata K. Takahashi M. Matsuba Y. Okuyama-Uchimura F. Sato K. Hashimoto S.. (2018). Generation of app knock-in mice reveals deletion mutations protective against Alzheimer’s disease-like pathology. Nat. Commun. 9:1800. doi: 10.1038/s41467-018-04238-0, PMID: 29728560 PMC5935712

[ref119] Nagpal R. Neth B. J. Wang S. Craft S. Yadav H. (2019). Modified Mediterranean-ketogenic diet modulates gut microbiome and short-chain fatty acids in association with Alzheimer’s disease markers in subjects with mild cognitive impairment. EBioMedicine 47, 529–542. doi: 10.1016/j.ebiom.2019.08.032, PMID: 31477562 PMC6796564

[ref120] Natale G. Biagioni F. Busceti C. L. Gambardella S. Limanaqi F. Fornai F. (2019). TREM receptors connecting bowel inflammation to neurodegenerative disorders. Cells 8:1124. doi: 10.3390/cells8101124 31546668 PMC6829526

[ref121] Oleskin A. V. Shenderov B. A. (2016). Neuromodulatory effects and targets of the SCFAs and gasotransmitters produced by the human symbiotic microbiota. Microb. Ecol. Health Dis. 27:30971. doi: 10.3402/mehd.v27.30971 27389418 PMC4937721

[ref122] Olsen I. Yamazaki K. (2019). Can oral bacteria affect the microbiome of the gut? J. Oral Microbiol. 11:1586422. doi: 10.1080/20002297.2019.1586422, PMID: 30911359 PMC6427756

[ref123] Ong I. M. Gonzalez J. G. McIlwain S. J. Sawin E. A. Schoen A. J. Adluru N.. (2018). Gut microbiome populations are associated with structure-specific changes in white matter architecture. Transl. Psychiatry 8:6. doi: 10.1038/s41398-017-0022-5, PMID: 29317592 PMC5802560

[ref124] Otero-Garcia M. Mahajani S. U. Wakhloo D. Tang W. Xue Y. Q. Morabito S.. (2022). Molecular signatures underlying neurofibrillary tangle susceptibility in Alzheimer’s disease. Neuron 110, 2929–48.e8. doi: 10.1016/j.neuron.2022.06.021 35882228 PMC9509477

[ref125] Pardridge W. M. Kang Y. S. Buciak J. L. (1994). Transport of human recombinant brain-derived neurotrophic factor (BDNF) through the rat blood-brain barrier in vivo using vector-mediated peptide drug delivery. Pharm. Res. 11, 738–746. doi: 10.1023/A:1018940732550 8058646

[ref126] Park J. C. Lim H. Byun M. S. Yi D. Byeon G. Jung G.. (2023). Sex differences in the progression of glucose metabolism dysfunction in Alzheimer’s disease. Exp. Mol. Med. 55, 1023–1032. doi: 10.1038/s12276-023-00993-3 37121979 PMC10238450

[ref127] Park H. Oh J. Shim G. Cho B. Chang Y. Kim S.. (2019). In vivo neuronal gene editing via CRISPR-Cas9 amphiphilic nanocomplexes alleviates deficits in mouse models of Alzheimer’s disease. Nat. Neurosci. 22, 524–528. doi: 10.1038/s41593-019-0352-0, PMID: 30858603

[ref128] Plascencia-Villa G. Perry G. (2021). Preventive and therapeutic strategies in Alzheimer’s disease: focus on oxidative stress, redox metals, and Ferroptosis. Antioxid. Redox Signal. 34, 591–610. doi: 10.1089/ars.2020.8134, PMID: 32486897 PMC8098758

[ref129] Prasad K. N. (2017). Oxidative stress and pro-inflammatory cytokines may act as one of the signals for regulating microRNAs expression in Alzheimer’s disease. Mech. Ageing Dev. 162, 63–71. doi: 10.1016/j.mad.2016.12.003, PMID: 27964992

[ref130] Qiu S. Joshi P. S. Miller M. I. Xue C. Zhou X. Karjadi C.. (2020). Development and validation of an interpretable deep learning framework for Alzheimer’s disease classification. Brain 143, 1920–1933. doi: 10.1093/brain/awaa137 32357201 PMC7296847

[ref131] Rafii M. S. Baumann T. L. Bakay R. A. Ostrove J. M. Siffert J. Fleisher A. S.. (2014). A phase1 study of stereotactic gene delivery of AAV2-NGF for Alzheimer’s disease. Alzheimers Dement. 10, 571–581. doi: 10.1016/j.jalz.2013.09.004, PMID: 24411134

[ref132] Rafii M. S. Tuszynski M. H. Thomas R. G. Barba D. Brewer J. B. Rissman R. A.. (2018). Adeno-associated viral vector (serotype 2)-nerve growth factor for patients with Alzheimer disease: a randomized clinical trial. JAMA Neurol. 75, 834–841. doi: 10.1001/jamaneurol.2018.0233, PMID: 29582053 PMC5885277

[ref133] Rahman M. M. Islam F. Or-Rashid M. H. Mamun A. A. Rahaman M. S. Islam M. M.. (2022). The gut microbiota (microbiome) in cardiovascular disease and its therapeutic regulation. Front. Cell. Infect. Microbiol. 12:903570. doi: 10.3389/fcimb.2022.903570, PMID: 35795187 PMC9251340

[ref134] Rahman A. Schelbaum E. Hoffman K. Diaz I. Hristov H. Andrews R.. (2020). Sex-driven modifiers of Alzheimer risk: a multimodality brain imaging study. Neurology 95, e166–e178. doi: 10.1212/WNL.0000000000009781, PMID: 32580974 PMC7455325

[ref135] Rashid T. Abdulkadir A. Nasrallah I. M. Ware J. B. Liu H. Spincemaille P.. (2021). DEEPMIR: a deep neural network for differential detection of cerebral microbleeds and iron deposits in MRI. Sci. Rep. 11:14124. doi: 10.1038/s41598-021-93427-x 34238951 PMC8266884

[ref136] Ravelli K. G. Rosário B. D. Camarini R. Hernandes M. S. Britto L. R. (2017). Intracerebroventricular Streptozotocin as a model of Alzheimer’s disease: neurochemical and behavioral characterization in mice. Neurotox. Res. 31, 327–333. doi: 10.1007/s12640-016-9684-7 27913964

[ref137] Ren J. Chen Y. I. Liu C. H. Chen P. C. Prentice H. Wu J. Y.. (2016). Non-invasive tracking of gene transcript and neuroprotection after gene therapy. Gene Ther. 23, 1–9. doi: 10.1038/gt.2015.81, PMID: 26207935 PMC4706495

[ref138] Ren P. Ding W. Li S. Liu G. Luo M. Zhou W.. (2023). Regional transcriptional vulnerability to basal forebrain functional dysconnectivity in mild cognitive impairment patients. Neurobiol. Dis. 177:105983. doi: 10.1016/j.nbd.2022.105983, PMID: 36586468

[ref139] Richardson R. M. Kells A. P. Martin A. J. Larson P. S. Starr P. A. Piferi P. G.. (2011). Novel platform for MRI-guided convection-enhanced delivery of therapeutics: preclinical validation in non-human primate brain. Stereotact. Funct. Neurosurg. 89, 141–151. doi: 10.1159/000323544, PMID: 21494065 PMC3085040

[ref140] Richardson R. M. Kells A. P. Rosenbluth K. H. Salegio E. A. Fiandaca M. S. Larson P. S.. (2011). Interventional MRI-guided putaminal delivery of AAV2-GDNF for a planned clinical trial in Parkinson’s disease. Mol. Ther. 19, 1048–1057. doi: 10.1038/mt.2011.11, PMID: 21343917 PMC3129792

[ref141] Risacher S. L. Saykin A. J. (2021). Neuroimaging advances in neurologic and neurodegenerative diseases. Neurotherapeutics 18, 659–660. doi: 10.1007/s13311-021-01105-7, PMID: 34410634 PMC8423931

[ref142] Rosario D. Bidkhori G. Lee S. Bedarf J. Hildebrand F. Le Chatelier E.. (2021). Systematic analysis of gut microbiome reveals the role of bacterial folate and homocysteine metabolism in Parkinson’s disease. Cell Rep. 34:108807. doi: 10.1016/j.celrep.2021.108807, PMID: 33657381

[ref143] Ruan D. Sun L. (2023). Amyloid-β PET in Alzheimer’s disease: a systematic review and Bayesian meta-analysis. Brain Behav. 13:e2850. doi: 10.1002/brb3.2850, PMID: 36573329 PMC9847612

[ref144] Sala A. Caminiti S. P. Presotto L. Pilotto A. Liguori C. Chiaravalloti A.. (2021). In vivo human molecular neuroimaging of dopaminergic vulnerability along the Alzheimer’s disease phases. Alzheimers Res. Ther. 13:187. doi: 10.1186/s13195-021-00925-1, PMID: 34772450 PMC8588696

[ref145] Scheltens P. De Strooper B. Kivipelto M. Holstege H. Chételat G. Teunissen C. E.. (2021). Alzheimer’s disease. Lancet 397, 1577–1590. doi: 10.1016/S0140-6736(20)32205-4 33667416 PMC8354300

[ref146] Schilling K. G. Archer D. Yeh F. C. Rheault F. Cai L. Y. Hansen C.. (2022). Aging and white matter microstructure and macrostructure: a longitudinal multi-site diffusion MRI study of 1218 participants. Brain Struct. Funct. 227, 2111–2125. doi: 10.1007/s00429-022-02503-z, PMID: 35604444 PMC9648053

[ref147] Sheline Y. I. Raichle M. E. (2013). Resting state functional connectivity in preclinical Alzheimer’s disease. Biol. Psychiatry 74, 340–347. doi: 10.1016/j.biopsych.2012.11.028 23290495 PMC3537262

[ref148] Sheng J. Zhang Q. Zhang Q. Wang L. Yang Z. Xin Y.. (2024). A hybrid multimodal machine learning model for detecting Alzheimer’s disease. Comput. Biol. Med. 170:108035. doi: 10.1016/j.compbiomed.2024.108035, PMID: 38325214

[ref149] Shi Z. Li H. Song W. Zhou Z. Li Z. Zhang M. (2023). Emerging roles of the gut microbiota in cancer immunotherapy. Front. Immunol. 14:1139821. doi: 10.3389/fimmu.2023.1139821, PMID: 36911704 PMC9992551

[ref150] Singer O. Marr R. A. Rockenstein E. Crews L. Coufal N. G. Gage F. H.. (2005). Targeting BACE1 with siRNAs ameliorates Alzheimer disease neuropathology in a transgenic model. Nat. Neurosci. 8, 1343–1349. doi: 10.1038/nn1531, PMID: 16136043

[ref151] Sone D. Shigemoto Y. Ogawa M. Maikusa N. Okita K. Takano H.. (2020). Association between neurite metrics and tau/inflammatory pathology in Alzheimer’s disease. Alzheimers Dement 12:e12125. doi: 10.1002/dad2.12125, PMID: 33204813 PMC7656172

[ref152] Su F. Bai F. Zhang Z. (2016). Inflammatory cytokines and Alzheimer’s disease: a review from the perspective of genetic polymorphisms. Neurosci. Bull. 32, 469–480. doi: 10.1007/s12264-016-0055-4, PMID: 27568024 PMC5563762

[ref153] Sudhakar V. Richardson R. M. (2019). Gene therapy for neurodegenerative diseases. Neurotherapeutics 16, 166–175. doi: 10.1007/s13311-018-00694-0, PMID: 30542906 PMC6361055

[ref154] Sun J. Carlson-Stevermer J. Das U. Shen M. Delenclos M. Snead A. M.. (2019). CRISPR/Cas9 editing of APP C-terminus attenuates β-cleavage and promotes α-cleavage. Nat. Commun. 10:53. doi: 10.1038/s41467-018-07971-8 30604771 PMC6318289

[ref155] Sun J. Xu J. Ling Y. Wang F. Gong T. Yang C.. (2019). Fecal microbiota transplantation alleviated Alzheimer’s disease-like pathogenesis in APP/PS1 transgenic mice. Transl. Psychiatry 9:189. doi: 10.1038/s41398-019-0525-3, PMID: 31383855 PMC6683152

[ref156] Sur C. Kost J. Scott D. Adamczuk K. Fox N. C. Cummings J. L.. (2020). BACE inhibition causes rapid, regional, and non-progressive volume reduction in Alzheimer’s disease brain. Brain 143, 3816–3826. doi: 10.1093/brain/awaa332, PMID: 33253354 PMC8453290

[ref157] Tan A. H. Lim S. Y. Lang A. E. (2022). The microbiome-gut-brain axis in Parkinson disease – from basic research to the clinic. Nat. Rev. Neurol. 18, 476–495. doi: 10.1038/s41582-022-00681-2, PMID: 35750883

[ref158] Tang W. H. W. Bäckhed F. Landmesser U. Hazen S. L. (2019). Intestinal microbiota in cardiovascular health and disease: JACC state-of-the-art review. J. Am. Coll. Cardiol. 73, 2089–2105. doi: 10.1016/j.jacc.2019.03.024, PMID: 31023434 PMC6518422

[ref159] Taylor X. Clark I. M. Fitzgerald G. J. Oluoch H. Hole J. T. DeMattos R. B.. (2023). Amyloid-β (Aβ) immunotherapy induced microhemorrhages are associated with activated perivascular macrophages and peripheral monocyte recruitment in Alzheimer’s disease mice. Mol. Neurodegener. 18:59. doi: 10.1186/s13024-023-00649-w 37649100 PMC10469415

[ref160] Thams F. Kuzmina A. Backhaus M. Li S. C. Grittner U. Antonenko D.. (2020). Cognitive training and brain stimulation in prodromal Alzheimer’s disease (AD-stim)-study protocol for a double-blind randomized controlled phase IIb (monocenter) trial. Alzheimers Res. Ther. 12:142. doi: 10.1186/s13195-020-00692-5, PMID: 33160420 PMC7648990

[ref161] Thompson T. (2024). How CRISPR gene editing could help treat Alzheimer’s. Nature 625, 13–14. doi: 10.1038/d41586-023-03931-5, PMID: 38082131

[ref162] Tiso M. Schechter A. N. (2015). Nitrate reduction to nitrite, nitric oxide and ammonia by gut bacteria under physiological conditions. PLoS One 10:e0119712. doi: 10.1371/journal.pone.0119712, PMID: 25803049 PMC4372352

[ref163] Tripathi S. M. Murray A. D. (2022). Alzheimer’s dementia: the emerging role of positron emission tomography. Neuroscientist 28, 507–519. doi: 10.1177/1073858421997035, PMID: 33660556 PMC9449436

[ref164] Upadhyay N. Suppa A. Piattella M. C. Bologna M. Di Stasio F. Formica A.. (2016). MRI gray and white matter measures in progressive supranuclear palsy and corticobasal syndrome. J. Neurol. 263, 2022–2031. doi: 10.1007/s00415-016-8224-y, PMID: 27411806

[ref165] van den Brink A. C. Brouwer-Brolsma E. M. Berendsen A. A. M. van de Rest O. (2019). The Mediterranean, dietary approaches to stop hypertension (DASH), and Mediterranean-DASH intervention for neurodegenerative delay (MIND) diets are associated with less cognitive decline and a lower risk of Alzheimer’s disease-a review. Adv. Nutr 10, 1040–1065. doi: 10.1093/advances/nmz054, PMID: 31209456 PMC6855954

[ref166] Varenika V. Dickinson P. Bringas J. LeCouteur R. Higgins R. Park J.. (2008). Detection of infusate leakage in the brain using real-time imaging of convection-enhanced delivery. J. Neurosurg. 109, 874–880. doi: 10.3171/JNS/2008/109/11/0874 18976077 PMC2725182

[ref167] Veale T. Malone I. B. Poole T. Parker T. D. Slattery C. F. Paterson R. W.. (2021). Loss and dispersion of superficial white matter in Alzheimer’s disease: a diffusion MRI study. Brain Commun 3:272. doi: 10.1093/braincomms/fcab272 PMC8633427 34859218

[ref168] Wagatsuma K. Miwa K. Akamatsu G. Yamao T. Kamitaka Y. Sakurai M.. (2023). Toward standardization of tau PET imaging corresponding to various tau PET tracers: a multicenter phantom study. Ann. Nucl. Med. 37, 494–503. doi: 10.1007/s12149-023-01847-8, PMID: 37243882

[ref169] Wahlström A. Sayin S. I. Marschall H. U. Bäckhed F. (2016). Intestinal crosstalk between bile acids and microbiota and its impact on host metabolism. Cell Metab. 24, 41–50. doi: 10.1016/j.cmet.2016.05.005, PMID: 27320064

[ref170] Wang T. Hu X. Liang S. Li W. Wu X. Wang L.. (2015). *Lactobacillus fermentum* NS9 restores the antibiotic induced physiological and psychological abnormalities in rats. Benefic. Microbes 6, 707–717. doi: 10.3920/BM2014.0177 25869281

[ref171] Wang J. Jin C. Zhou J. Zhou R. Tian M. Lee H. J.. (2023). PET molecular imaging for pathophysiological visualization in Alzheimer’s disease. Eur. J. Nucl. Med. Mol. Imaging 50, 765–783. doi: 10.1007/s00259-022-05999-z, PMID: 36372804 PMC9852140

[ref172] Wang H. Shang Y. Wang E. Xu X. Zhang Q. Qian C.. (2022). MST1 mediates neuronal loss and cognitive deficits: a novel therapeutic target for Alzheimer’s disease. Prog. Neurobiol. 214:102280. doi: 10.1016/j.pneurobio.2022.102280 35525373

[ref173] Wang Q. H. Wang Y. R. Zhang T. Jiao S. S. Liu Y. H. Zeng F.. (2016). Intramuscular delivery of p75NTR ectodomain by an AAV vector attenuates cognitive deficits and Alzheimer’s disease-like pathologies in APP/PS1 transgenic mice. J. Neurochem. 138, 163–173. doi: 10.1111/jnc.13616, PMID: 26991827

[ref174] Wareham L. K. Liddelow S. A. Temple S. Benowitz L. I. Di Polo A. Wellington C.. (2022). Solving neurodegeneration: common mechanisms and strategies for new treatments. Mol. Neurodegener. 17:23. doi: 10.1186/s13024-022-00524-0, PMID: 35313950 PMC8935795

[ref175] Xiang X. Wind K. Wiedemann T. Blume T. Shi Y. Briel N.. (2021). Microglial activation states drive glucose uptake and FDG-PET alterations in neurodegenerative diseases. Sci. Transl. Med. 13:eabe5640. doi: 10.1126/scitranslmed.abe5640 34644146

[ref176] Xiao L. Yang X. Sharma V. K. Abebe D. Loh Y. P. (2023). Hippocampal delivery of neurotrophic factor-α1/carboxypeptidase E gene prevents neurodegeneration, amyloidosis, memory loss in Alzheimer’s disease male mice. Mol. Psychiatry 28, 3332–3342. doi: 10.1038/s41380-023-02135-7, PMID: 37369719 PMC10618095

[ref177] Xu Q. Q. Su Z. R. Yang W. Zhong M. Xian Y. F. Lin Z. X. (2023). Patchouli alcohol attenuates the cognitive deficits in a transgenic mouse model of Alzheimer’s disease via modulating neuropathology and gut microbiota through suppressing C/EBPβ/AEP pathway. J. Neuroinflammation 20:19. doi: 10.1186/s12974-023-02704-1, PMID: 36717922 PMC9887791

[ref178] Yang W. Pilozzi A. Huang X. (2021). An overview of ICA/BSS-based application to Alzheimer’s brain signal processing. Biomedicines 9:386. doi: 10.3390/biomedicines9040386, PMID: 33917280 PMC8067382

[ref179] Yang Y. Tapias V. Acosta D. Xu H. Chen H. Bhawal R.. (2022). Altered succinylation of mitochondrial proteins, APP and tau in Alzheimer’s disease. Nat. Commun. 13:159. doi: 10.1038/s41467-021-27572-2, PMID: 35013160 PMC8748865

[ref180] Yang G. Wei J. Liu P. Zhang Q. Tian Y. Hou G.. (2021). Role of the gut microbiota in type 2 diabetes and related diseases. Metab. Clin. Exp. 117:154712. doi: 10.1016/j.metabol.2021.154712 33497712

[ref181] Yeh Y. C. Li C. W. Kuo Y. T. Huang M. F. Liu T. L. Jaw T. S.. (2018). Association between altered neurochemical metabolites and apathy in patients with Alzheimer’s disease. Int. Psychogeriatr. 30, 761–768. doi: 10.1017/S1041610217002381 29143702

[ref182] Yoo T. J. (2022). Anti-inflammatory gene therapy improves spatial memory performance in a mouse model of Alzheimer’s disease. J. Alzheimers Dis. 85, 1001–1008. doi: 10.3233/JAD-215270, PMID: 34897091 PMC8925118

[ref183] Yulug B. Hanoglu L. Ozansoy M. Isık D. Kilic U. Kilic E.. (2018). Therapeutic role of rifampicin in Alzheimer’s disease. Psychiatry Clin. Neurosci. 72, 152–159. doi: 10.1111/pcn.12637 29315976

[ref184] Zhang Y. Liu S. (2018). Analysis of structural brain MRI and multi-parameter classification for Alzheimer’s disease. Biomed Tech (Berl) 63, 427–437. doi: 10.1515/bmt-2016-0239 28622141

[ref185] ZhangX. X.TianY.WangZ. T.MaY. H.TanL.YuJ. T. (2021). The epidemiology of Alzheimer’s disease modifiable risk factors and prevention. J. Prev Alzheimers Dis. 8, 313–321. doi: 10.14283/jpad.2021.15, PMID: 34101789

[ref186] Zhang M. Zhu W. Ma Y. Huang K. Huang S. Chen Q.. (2021). Early neurological deterioration and Hypoperfusion volume ratio on arterial spin labeling in patients with acute ischemic stroke. J. Stroke Cerebrovasc. Dis. 30:105885. doi: 10.1016/j.jstrokecerebrovasdis.2021.105885 34107416

[ref187] Zhao Y. Alexandrov P. N. Lukiw W. J. (2016). Anti-microRNAs as novel therapeutic agents in the clinical Management of Alzheimer’s disease. Front. Neurosci. 10:59. doi: 10.3389/fnins.2016.00059 26941600 PMC4766517

[ref188] Zhao J. Fu Y. Yamazaki Y. Ren Y. Davis M. D. Liu C. C.. (2020). APOE4 exacerbates synapse loss and neurodegeneration in Alzheimer’s disease patient iPSC-derived cerebral organoids. Nat. Commun. 11:5540. doi: 10.1038/s41467-020-19264-0, PMID: 33139712 PMC7608683

[ref189] Zhao Y. Li T. Zhu H. (2022). Bayesian sparse heritability analysis with high-dimensional neuroimaging phenotypes. Biostatistics 23, 467–484. doi: 10.1093/biostatistics/kxaa035 32948880 PMC9308456

[ref190] Zuccato C. Cattaneo E. (2009). Brain-derived neurotrophic factor in neurodegenerative diseases. Nat. Rev. Neurol. 5, 311–322. doi: 10.1038/nrneurol.2009.54 19498435

